# Regulating Promiscuous Catalysis via Substrate‐Induced Transient Assembly

**DOI:** 10.1002/anie.202511352

**Published:** 2025-10-16

**Authors:** Ayan Chatterjee, Maximilian Schuler, Marius G. Braun, Christopher V. Synatschke, Qi Lu, Jiyao Yu, David Y.W. Ng, Tanja Weil

**Affiliations:** ^1^ Max Planck Institute for Polymer Research Ackermannweg 10 D‐55128 Mainz Germany; ^2^ Max Planck School Matter to Life Jahnstraße 29 D‐69120 Heidelberg Germany

**Keywords:** Carbamate cleavage, Emergent catalysis, Nonequilibrium assembly, Peptides, Promiscuity

## Abstract

In nature, substrate‐induced assembly is a fundamental requirement for a wide range of enzyme‐driven chemical transformations. Systems chemists have introduced synthetic substrate analogues that have proven effective in enhancing catalytic activities of assembling peptide folds, and mimic primitive enzymes. However, how catalytic promiscuity, the ability of one catalyst to catalyse multiple orthogonal reactions, might have shaped the diversification of prebiotic chemistry, remains largely unaddressed. Herein, we report a novel transient, substrate‐induced co‐assembly between a lysine‐rich pre‐assembling peptide and Fmoc‐glycine. The nanostructure formed under nonequilibrium conditions provides the suitable microenvironment to promote the potential of catalytically active amino acids, performing orthogonal hydrolysis and C═N condensation reactions. Simultaneously, carbamate bond cleavage of the labile Fmoc group destabilises the co‐assembly in the activated state, causing the structure to collapse gradually. By encoding catalytic promiscuity into assembling building blocks under kinetic control, we shed light on the emergence of primitive catalysis with broad substrate scope at the origin of life.

## Graphical abstract

We report a novel substrate‐induced transient co‐assembly between a short Lys‐rich peptide and Fmoc‐Gly‐OH. In the activated state, the assembly exhibits promiscuous catalytic activity through the rate enhancements of hydrolysis and C═N condensation reactions. Over time, the substrate Fmoc‐Gly‐OH is cleaved into smaller fragments, leading to the collapse of the hydrogel and a decrease in catalytic activity.

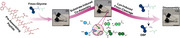

In living organisms, polymerisation of biomolecules and precise positioning of active residues across three‐dimensional space are crucial for a diverse range of functional capabilities, such as emergence of catalytic reaction networks, transport highways and formation of complex phase‐separated states.^[^ ^1^, ^2^, ^3^, ^4^ ^]^ These systems are often installed with regulatory steps through a feedback process, sustained by energy sources in the form of substrates under out‐of‐equilibrium conditions. In particular, cytoskeletal proteins such as actin and tubulin bind nucleotides on the monomeric level and hydrolyse them in the assembled state, leading to transient supramolecular polymerisation.^[^ ^5^, ^6^ ^]^ These dynamic properties are indispensable for a diverse set of biological functions, for instance, moving payloads, cell division, motility and so forth.^[^ ^7^, ^8^ ^]^ Importantly, catalytic traits of the assembled biopolymer are steered by the underlying chemical reaction network, which determines its lifetime kinetically.^[^ ^9^, ^10^, ^11^ ^]^ Additionally, such transient supramolecular architectures are often observed for large scale protein complexes, which unleash diverse sets of reaction networks in a spatiotemporal manner.^[^ ^12^ ^]^

Inspired from such biological complexities, the remit of chemistry has recently expanded towards focusing on dynamic and functional soft nanostructures that emanate from multicomponent biomolecular crowding and seek to bridge the gap between ′dead′ and living matter.^[^ ^13^, ^14^, ^15^, ^16^, ^17^, ^18^, ^19^, ^20^, ^21^, ^22^, ^23^, ^24^, ^25^, ^26^, ^27^, ^28^, ^29^, ^30^, ^31^, ^32^, ^33^, ^34^, ^35^, ^36^, ^37^, ^38^, ^39^, ^40^, ^41^, ^42^, ^43^, ^44^ ^]^ Importantly, phylogenetic studies have suggested primitive peptide‐based polymers as key players for the onset of metabolism, a crucial path towards shaping primitive living system.^[^ ^34^, ^39^, ^40^, ^41^ ^]^ By fostering a series of orthogonal chemical transformations, they could have enriched the prebiotic chemical toolbox, a precursor towards emergence of proto‐metabolic reaction networks. Moreover, based on environmental and chemical cues and by promoting promiscuous chemical transformations, such minimalistic peptides could have been crucial in filling the gap between chemical building blocks and self‐replicating systems, a step necessary for the evolution of more complex and efficient biological systems.^[^ ^40^, ^41^, ^42^, ^43^, ^44^ ^]^

To this end, systems chemists have developed a plethora of synthetic chemical reaction networks based on assembling peptides or other small molecular precursors that mimic the nonequilibrium characteristics as abundantly observed in nature.^[^ ^37^, ^45^, ^46^, ^47^, ^48^, ^49^, ^50^, ^51^, ^52^, ^53^, ^54^, ^55^, ^56^, ^57^, ^58^, ^59^, ^60^, ^61^, ^62^, ^63^ ^]^ However, diversity of new reaction cycles and their role as promiscuous systems in the scope of chemical evolution and a step towards designing next generation smart materials has barely been touched.

In this direction, we here report a novel approach to substrate‐driven transient assembly, featuring temporal catalytically promiscuous states by combining Fmoc‐protected glycine (**Fmoc‐Gly**) and a short pre‐assembling peptide Fmoc‐HLRLKLK‐NH_2_ (**P1**) (Figures 1 and S1). We hypothesised that due to its highly charged nature given by Arg and Lys residues, the peptide would not self‐assemble by itself at pH 8. Only when subjected to **Fmoc‐Gly**, the π–π interactions between **Fmoc‐Gly** and **P1** would mediate the charges and trigger a co‐assembly leading to a supramolecular architecture. In this activated state, His and Lys residues could then unfold their catalytic potential to promote hydrolysis and C═N condensation reactions due to the suitable microenvironment provided by the assembly. Over time, the Brønsted‐base property of Lys in the co‐assembly could then take over to cleave the kinetically stable yet thermodynamically activated weak carbamate bond between the Fmoc group and the Gly unit.^[^ ^64^ ^]^ This Lys‐induced cleavage of **Fmoc‐Gly** into dibenzofulvene (DBF), Gly and CO_2_ would then drive subsequent disassembly into catalytically less active fragments (Figure 1). With this work, we go beyond existing contributions as we, for the first time, report a three‐fold catalytic promiscuity that not only covers catalysis under nonequilibrium conditions but also a broad substrate and reaction diversity.

**Figure 1:**
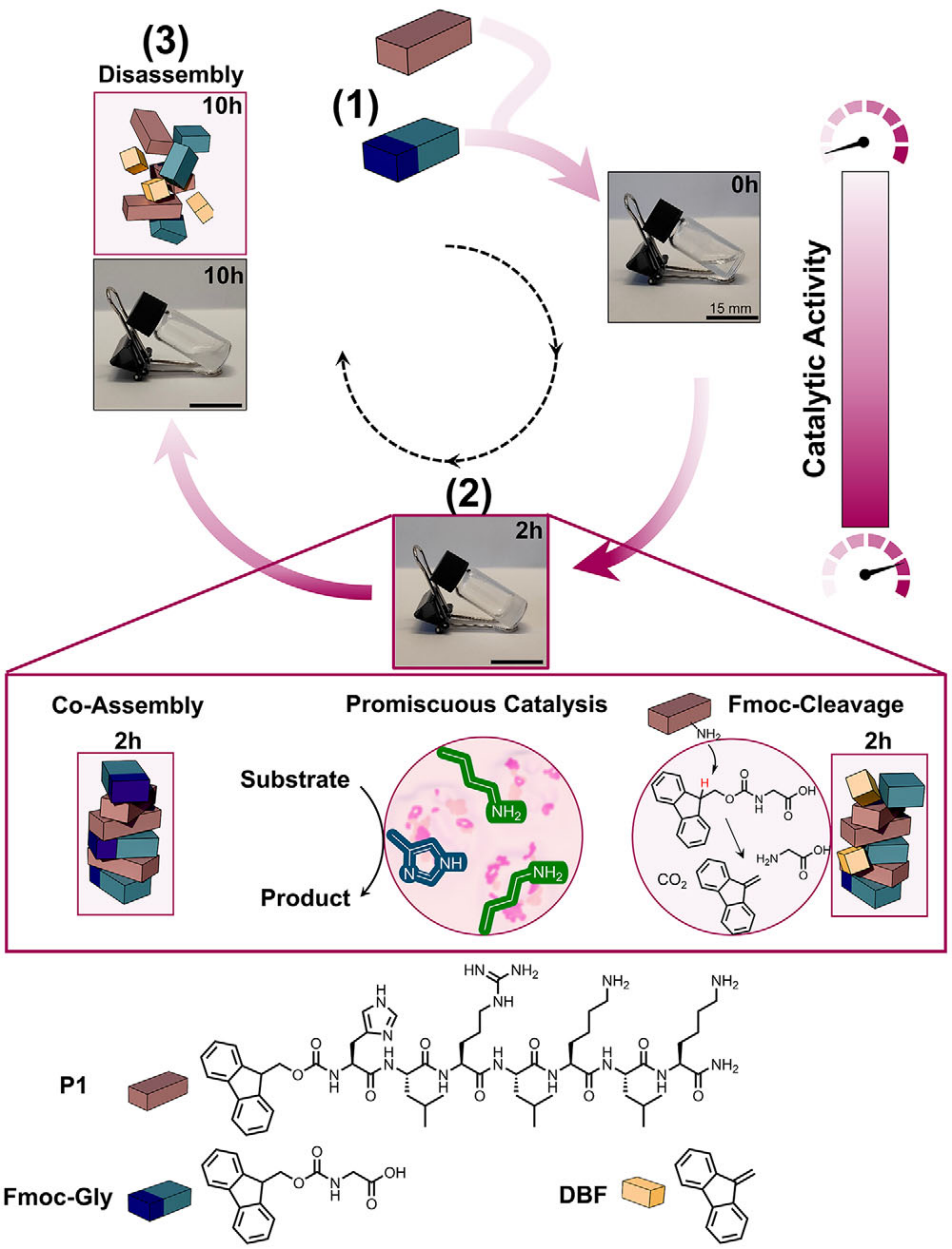
Schematic illustration of substrate‐induced catalytic reaction cycle. **1**) Soluble **P1** and **Fmoc‐Gly** show low catalytic activity in the sol state. **2**) Over time **P1** and **Fmoc‐Gly** co‐assemble to a self‐standing hydrogel in which catalytically active amino acids perform multiple chemical transformations showing catalytic promiscuity. In the assembly, Lys residues of **P1** catalyse the **Fmoc‐Gly** cleavage into DBF. **3**) The destabilisation leads to the disassembly of nanofibres, followed by a macroscopic gel‐sol transition.

We first probed the ability of **Fmoc‐Gly** to induce the assembly of **P1** by initial screening of various molar ratios at constant buffer conditions (20% v/v DMSO, HEPES 100 mM, pH 8) (Figure 2a). Interestingly, when mixed equimolarly (2.5 mM each), the **P1**/**Fmoc‐Gly** mixture quickly became viscous and formed a self‐supporting gel, with maximum stability observed after 2 h (Figures 2b and S2 for a detailed phase diagram). Furthermore, a gradual weakening of the gel leading to a sol transition (or DBF precipitate) was observed after 10 h (Figures 2b and S3). We chose this condition as it lies on the phase boundary and therefore exhibited dynamic characteristics. Increased or decreased concentrations of both **P1** or **Fmoc‐Gly** either lacked the ability to form gels or to dissipate within 10 h (Figure S2). As a control, **P1** or **Fmoc‐Gly** alone formed only few amorphous, nonspecific aggregates, unable to exhibit comparable viscous characteristics (Figure S4a,b). The rheological properties were probed, and the **P1**/**Fmoc‐Gly** gel exhibited a transient change in storage modulus (G’) peaking at approx. 2 h (1905.66 ± 395.82 Pa) showing more than one order of magnitude difference compared to the initial and final states (Figure 2c). In stark contrast, **P1** or **Fmoc‐Gly** alone showed marginal storage modulus values (<G’ of 20 Pa), indicating a liquid‐like phase behaviour (Figure 2d). Intrigued by the bulk properties of the system, we went on to investigate the supramolecular architecture. The fluorescent dye Nile Red (NR) is known to interact with hydrophobic structures like amyloidal fibrils, changing its emission maxima in the bound state.^[^ ^63^ ^]^ Interestingly, for the system, a shift from 661 to 629 nm after 2 h was detected (Figure S5). However, this shift followed a transient pattern, indicating formation of nanostructures and subsequent disassembly. Confocal laser scanning microscopy (CLSM) corroborated these observations showing dense fibre formation and dissolution into aggregated structures after 2 h and 10 h, respectively (Figure 2e). Notably, for the individual components **Fmoc‐Gly** and **P1**, fibre formation remained completely absent as confirmed by transmission electron microscopy (TEM) and CLSM (Figures S4 c,d and S6). Furthermore, the turbidity monitored by visible light scattering (*λ*  = 600 nm) showed a substantial change over time for the **P1**/**Fmoc‐Gly** system. A strong increase in scattering intensity from 0.05 ± 0.03 to 0.63 ± 0.05 (ca. 3.5 h) was followed by a decrease to 0.56 ± 0.05 after 10 h (Figure S7). We hypothesise that the turbid nature of DBF precipitates hinders the system from turning completely transparent. Nevertheless, for **Fmoc‐Gly** and **P1** individually, no significant changes of absorption (<0.1) were detected, indicating the lack of self‐assembled nanostructures (Figure S7). Consistent with the previous data, probing the system on the nanoscale by TEM and atomic force microscopy (AFM) provided congruent insights. Both methods confirmed no initial structure formation (0 h) followed by co‐assembly into nanofibres (2 h) and dissolution into amorphous nonspecific aggregates (10 h) originating from the collapse of the system (Figures 2f,g and S8).^[^ ^46^ ^]^

**Figure 2:**
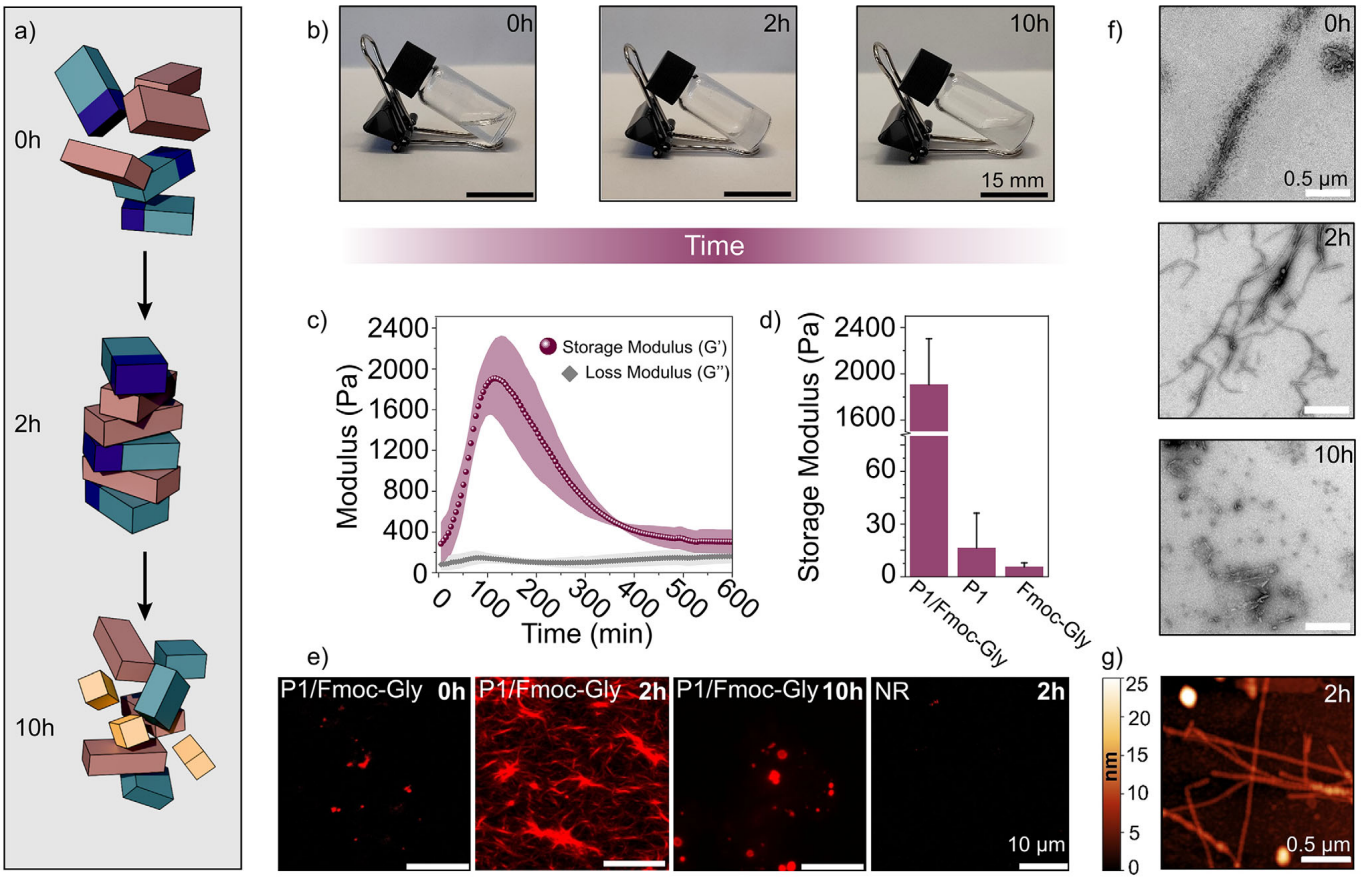
a) Illustration of the transient assembly comprising **P1** and **Fmoc‐Gly**. Assembly (2 h) is followed by subsequent cleavage of **Fmoc‐Gly** units (10 h) triggering the disassembly of the system. b) Representative phase transitions in a temporal fashion at different time points. c) Time‐dependent change of viscoelastic property of **P1/Fmoc‐Gly** under constant strain (1%) and frequency (1 Hz). d) Storage modulus of **P1/Fmoc‐Gly**; **P1** and **Fmoc‐Gly** at approx. 2 h of incubation. e) Timedependent CLSM micrographs of NR bound **P1/Fmoc‐Gly** and control only NR. Time course of f) TEM and g) AFM micrograph (2 h) of the **P1/Fmoc‐Gly** system. All experiments were conducted in 100 mM HEPES, 20% v/v DMSO, pH 8 using 2.5 mM of **P1** and 2.5 mM of **Fmoc‐Gly**. Error bars represent standard deviations from triplicates (*n* = 3).

Motivated by these findings, we proceeded to investigate the underlying molecular mechanism causing the structure to collapse. We suspected the observed dynamicity emanated from the presence of supramolecular assemblies; where supporting Lys units at assembled state, could act as a base to cleave **Fmoc‐Gly** (Figure 3a). Indeed, we observed the generation of the product DBF in the **P1**/**Fmoc‐Gly** reaction mixture. Product formation was confirmed by high performance liquid chromatography (HPLC), showing the same retention time (*t*_R_ = 22.0 min) as the commercially purchased DBF (Figure S9). Further, ^1^H‐NMR of DBF confirmed the molecular structure and the positive control (piperidine‐treated **Fmoc‐Gly**) corroborated Fmoc‐cleavage into DBF (Figure S9). The HPLC kinetics showed a remarkable increase in DBF concentration and simultaneous decrease in **Fmoc‐Gly** (Figure 3b). After an initial lag phase a sudden burst (after 2 h) of **Fmoc‐Gly** conversion was observed underpinning the importance of the assembly in the cleavage mechanism (Figure 3c). At this time point, DBF is measured to be ∼10%, indicating no significant contribution towards co‐assembly and hydrogel formation. Correspondingly, **Fmoc‐Gly** was consumed up to 82.50 ± 0.78% after 10 h (Figure 3d). Furthermore, the controls of individual 2.5 mM **P1** and **Fmoc‐Gly** showed negligible product formation after 10 h (< 2%) (Figure 3d). It is important to note that, even though **P1** also exhibits an Fmoc unit, its signal remained stable throughout the whole experiment, confirming that self‐degradation does not occur (Figure S10). We hypothesise that selective intermolecular cleavage of **Fmoc‐Gly** occurs due to a highly concentrated phase inside the hydrogel, which favours intermolecular over intramolecular carbamate cleavage.^[^ ^65^, ^66^ ^]^ To further investigate the role of Lys‐rich assemblies towards the substrate, we introduced a control peptide Fmoc‐HLRLHLR‐NH_2_ (**P2**) (Figure 3a), lacking Lys while keeping other amino acid residues largely constant.

**Figure 3:**
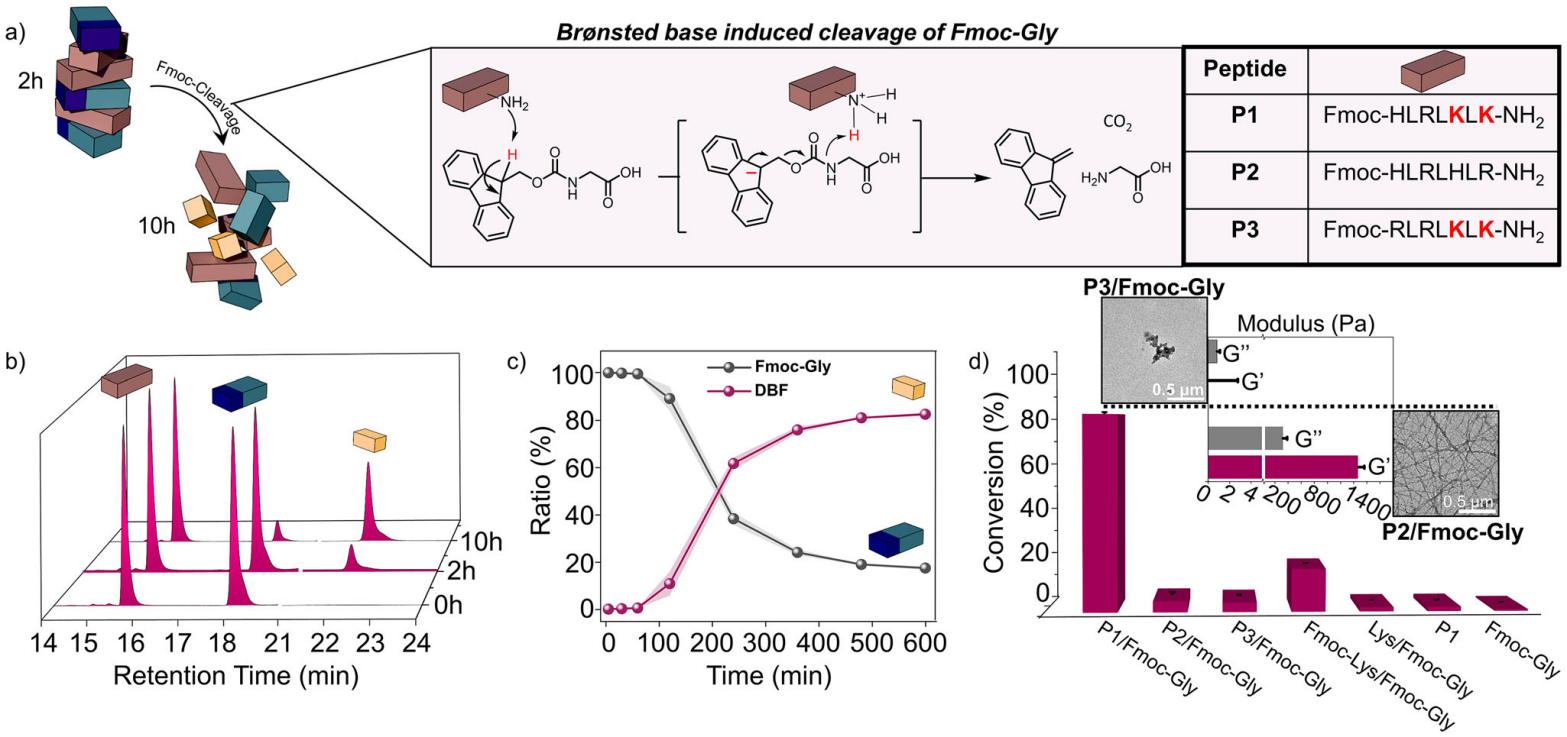
a) Tabular representation of carbamate bond cleavage of **Fmoc‐Gly** by peptide based transient assemblies. Primary amino acid sequences of **P1** and control peptides **P2** and **P3**. b) Time‐dependent HPLC traces (214 nm) of reaction mixture of **P1** and **Fmoc‐Gly**. c) HPLC kinetic study showing time course generation of DBF and consumption of **Fmoc‐Gly** for the system. d) Bar‐diagram based on HPLC data representing the relative **Fmoc‐Gly** conversion after 10 h for the system and respective controls; inset showing TEM micrograph and comparison of storage and loss modulus between **P2/Fmoc‐Gly** and **P3/Fmoc‐Gly** systems (both at 10 h). As **P3/Fmoc‐Gly** shows water‐like characteristics, no reliable G’ could be measured. Error bars represent standard deviations from triplicates (*n* = 3).

After the addition of **Fmoc‐Gly**, the formation of a highly viscous mixture with significant differences of storage and loss modulus (indicating gel characteristics) was observed. In presence of **Fmoc‐Gly**, the solution subsequently turned into a self‐supporting gel at 2 h, accompanied by a constant increase in storage modulus over time (Figures 3d and S11a,b). In stark contrast to the **P1**/**Fmoc‐Gly** system, **P2**/**Fmoc‐Gly** also remained stable after 16 h (Figure S11a). Notably, fibrous structures were formed after 2 h which persisted over time as confirmed by TEM (Figures 3d and S11c). Interestingly, marginal **Fmoc‐Gly** consumption was detected (4.70 ± 1.08% after 10 h), underpinning the importance of Lys residues to act as Brønsted bases in the cleavage mechanism of **Fmoc‐Gly** units (Figures 3a,d and S11d). To support the importance of assembly towards catalytic cleavage of **Fmoc‐Gly**, we further introduced a peptide sequence Fmoc‐RLRLKLK‐NH_2_ (**P3**) (Figures 3a and S1), which in the presence of **Fmoc‐Gly** did only form a few amorphous, nonspecific aggregates suggesting no ordered supramolecular structure formation (Figures 3d and S12a). Despite containing the equal number of Lys units with respect to **P1**, the **P3** could not demonstrate any significant catalytic cleavage of **Fmoc‐Gly** (4.04 ± 0.46% after 10 h) (Figures 3d and S12b). Of note, mixtures of **P3** and **Fmoc‐Gly** were unable to form any self‐supporting gel or significant viscous characteristics as observed for both the **P1**/**Fmoc‐Gly** and **P2**/**Fmoc‐Gly** systems (Figure S12c,d). Therefore, the results indicate the importance of supramolecular architecture and subsequent effect of proximal Lys residues at assembled states towards substrate consumptions. To further support this hypothesis, we subjected **Fmoc‐Gly** to twice the concentration (5 mM) of Fmoc‐Lys(NH_2_)‐OH (**Fmoc‐Lys**) or free H_2_N‐Lys‐OH (**Lys**) compared to **P1** to probe their **Fmoc‐Gly** cleavage efficacies. For the **Lys**/**Fmoc‐Gly** mixture, the formation of defined supramolecular architecture was absent after 10 h as confirmed by TEM (Figure S13a), whereas **Fmoc‐Lys**/**Fmoc‐Gly** samples formed wide nanotapes (Figure S13b). Correspondingly, **Lys**/**Fmoc‐Gly** failed to cleave significant amounts of **Fmoc‐Gly** (1.82 ± 0.02% after 10 h, Figure 3d). Interestingly, even though **Fmoc‐Lys**/**Fmoc‐Gly** co‐assembled, the **Fmoc‐Gly** conversion was only at 18.18 ± 0.12% and thus significantly lower as compared to the **P1**/**Fmoc‐Gly** system (Figure 3d). This highlights the importance of the molecular architecture surrounding the **Fmoc‐Gly**’s carbamate bond in the **P1**/**Fmoc‐Gly** system suggesting that **P1** provides superior scaffold properties as to simple building blocks like **Fmoc‐Lys**. To expand the substrate scope of the **P1**, we probed the system for different Fmoc‐substrates. Whereas the Fmoc cleavage efficiency was slightly decreased when **Fmoc‐Gly** was replaced by Fmoc‐Ala‐OH (**Fmoc‐Ala**) (61.26 ± 0.53% **Fmoc‐Ala** conversion after 10 h), it dropped significantly when Fmoc‐Ile‐OH (**Fmoc‐Ile**) was introduced instead (11.94 ± 0.01% **Fmoc‐Ile** conversion after 10 h) (Figure S14). Therefore, we hypothesise that highly sterically demanding amino acids like **Fmoc‐Ile** hinder Lys residues of **P1** to efficiently access the carbamate bond, slowing down the overall cleavage reaction. This observation demonstrates the sensitivity of the hydrolysis reaction on the Fmoc‐adjacent amino acid, with larger side chains inhibiting the cleavage. This result further supports the stability of **P1** as the sterical demand of His prevents its own hydrolysis.

Inspired by primordial biology, we wondered whether these peptide based catalytic folds under nonequilibrium dynamics were capable of driving multiple orthogonal chemical reactions, foreshadowing the evolutionary implications of prebiotic enzymes towards proto‐metabolism.^[^ ^41^, ^64^, ^67^ ^]^ It was anticipated that the transient nanofibres could provide the suitable microenvironment to drive promiscuous catalysis through His and Lys residues (Figure 4a). Notably, His and Lys residues are known to promote hydrolytic activity and condensation reactions, respectively.^[^ ^68^, ^69^, ^70^, ^71^, ^72^ ^]^ It is important to mention that even though Lys can in principle act as a nucleophile to promote ester hydrolysis, this effect is modest as compared to His at pH 8.^[^ ^68^, ^69^, ^70^, ^71^ ^]^ To probe the system for hydrolase‐like activity, the model compound 4‐nitrophenyl acetate (PNPA) was chosen (Figure 4b). This simple chromogenic substrate is widely known for the appearance of an intense product absorption band at 400 nm, stemming from the 4‐nitrophenol/‐ate. The addition of 100 µM of PNPA with the mixture of **P1**/**Fmoc‐Gly** at three different time intervals (0, 2 and 10 h) showed varied catalytic response (Figure 4c). The system at 2 h demonstrated ca. 5.5‐fold of initial rate enhancement (*V*_i_ = 6.13 ± 0.18 µM min^−1^) compared to the system at 0 h. The system at 2 h further hydrolyses PNPA with higher competencies (ca. 2.5 fold) compared to the 10 h time frame (Figures 4d and S15). This could be attributed to the presence of His containing peptide assemblies at 2 h compared to initial (0 h) and final stage (10 h) of the system (Figure 2f). Notably, marginal background hydrolysis of PNPA at only buffered solution (pH 8 HEPES 100 mM) was observed (Figure 4c,d). Using a more sterically hindered ester, *p*‐nitrophenyl butyrate (PNBA), the system also demonstrated transient hydrolytic capabilities as similarly observed for PNPA (Figure S16). However, due to the intrinsically higher stability, the system showed an activity of 3.63 ± 0.8 µM min^−1^ at 2 h and was thus approximately 1.7‐fold less efficient in hydrolysing PNBA as compared to PNPA. Nevertheless, it demonstrated more than one order of magnitude higher catalytic efficiency than the background hydrolysis (Figure S16).

**Figure 4:**
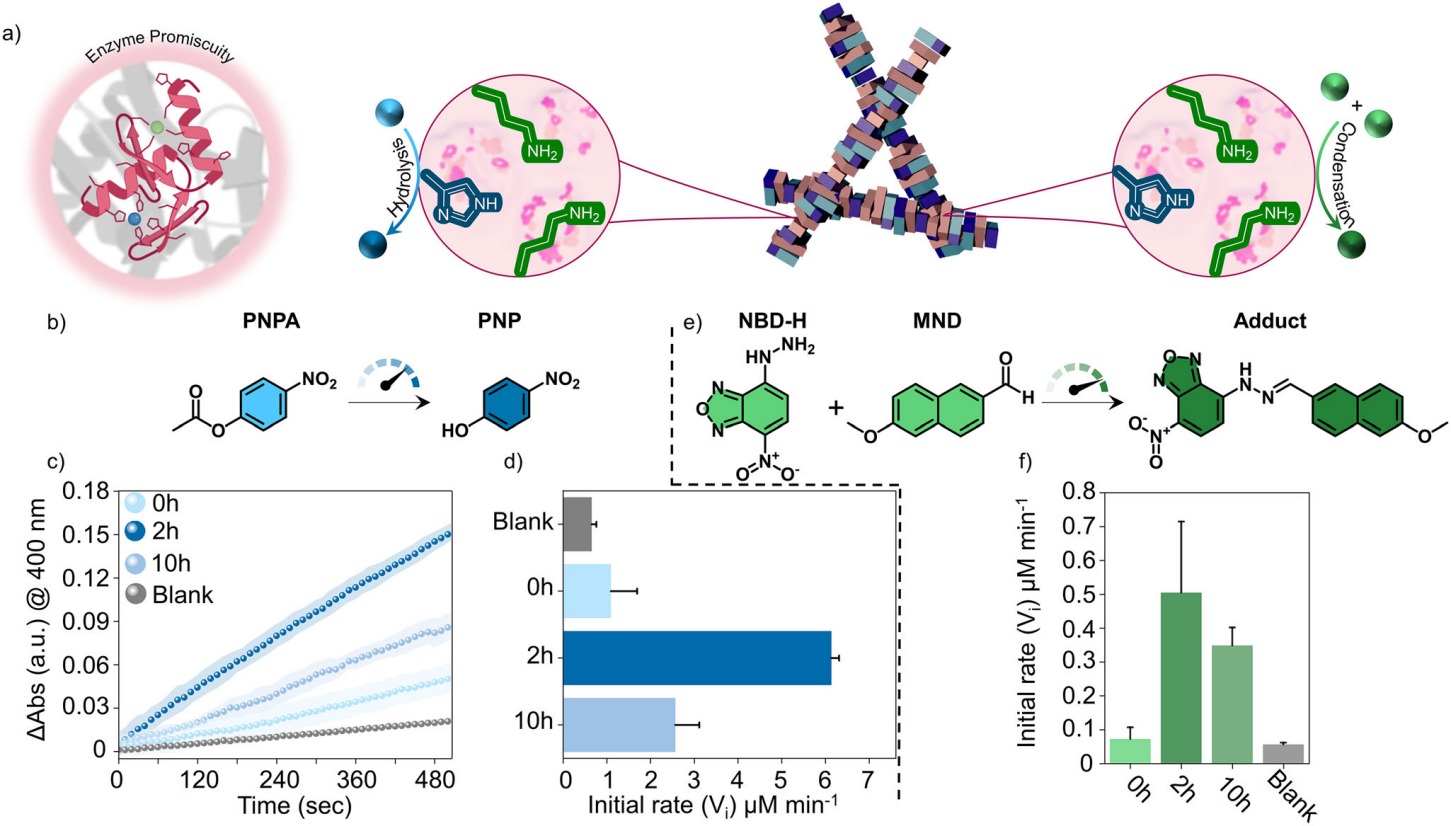
a) Schematic representation of accelerated catalytic promiscuity by transiently assembled supramolecular motifs. b) Model substrate to probe for rate enhancements of hydrolysis. c) Time course kinetic profiles of the system at different time intervals and d) the bar diagram showing the comparison of initial rates towards PNPA hydrolysis. e) Model C═N condensation reaction catalysed by the system. f) Comparison of catalytic abilities of system at different time intervals towards C═N condensation reaction. Error bars represent standard deviations from triplicates(n = 3). [**P1**/**Fmoc‐Gly**]: 0.5 mM each component (diluted from 2.5 mM). Figure 4a (left) was created by Schuler, M. (2025) in BioRender: https://BioRender.com/r8ygeqx.

Next, we investigated whether the Lys‐rich fibres could also feature the catalytic C═N condensation reaction between 4‐hydrazino‐7‐nitro‐2,1,3‐benzoxadiazole (NBD‐H) and 6‐methoxy‐2‐naphthaldehyde (MND) into the chromogenic hydrazone adduct (Figure 4e). Indeed, we observed an increase of absorption (at 524 nm) over time in presence of **P1**/**Fmoc‐Gly** upon addition of NBD‐H (0.2 mM) and MND (0.4 mM) (Figure S17). The initial rates for the 2 h system demonstrated almost one order of magnitude increase as compared to the background rates. Further, the system at 2 h showed the highest catalytic effect (0.50 ± 0.21 µM min^‐^^1^) compared to system at 0 h and 10 h (Figure 4f), suggesting a temporal regulation of catalytic promiscuity under nonequilibrium dynamics. Finally, we tested the impact of self‐assembly on catalytic efficiency for which soluble His (2.5 mM), Lys (5 mM) and Arg (2.5 mM) were chosen as controls for both hydrolysis and condensation reactions. The self‐assembled system at 2 h demonstrated a 2.6‐fold and 8.4‐fold higher activity for the hydrolysis and condensation reaction, respectively. This further emphasise the importance of the co‐assembled structures to impart catalytic activity (Figure S18).

In summary, the present work demonstrates a substrate‐induced out of equilibrium supramolecular co‐assembly between a short Lys‐rich peptide and Fmoc‐glycine molecules that could steer its own degradation via carbamate cleavage. Further, the encoded His and Lys residues of the peptide sequence have been exploited to unfold their promiscuous catalytic effect to promote hydrolysis and C═N condensation reactions, which emanated from the suitable microenvironment provided by the assembly. By combining catalytic diversity to spatiotemporal assembly–disassembly features, the present system foreshadows dynamicity of rudimentary peptides that could have fostered the enrichment of prebiotic chemical toolbox. Moreover, such minimalistic synthetic models offer a compelling framework for probing how transiently organised matter fuelled by environmental fluxes may have seeded the emergence of self‐sustaining chemical networks.

## Experimental Section

Experimental details in supporting information.

## Conflict of Interests

The authors declare no conflict of interest.

## Supporting information



Supporting Information

## Data Availability

The data that support the findings of this study are available in the Supporting Information of this article.
