# Supplementary material for: Regulating Promiscuous Catalysis via Substrate‐Induced Transient Assembly
Source: Angew Chem Int Ed Engl. 2025 Oct 16;65(1):e202511352. doi: 10.1002/anie.202511352 (PMC12759214; doi:10.1002/anie.202511352)
Supplement: Supplementary file 1 — Supporting Information [file ANIE-65-e202511352-s001.docx]

Supporting Information

**Regulating Promiscuous Catalysis via Substrate-Induced Transient Assembly**

Ayan Chatterjee,‡^[a]^ Maximilian Schuler,‡^[a][b]^ Marius G. Braun,^[a]^ Christopher V. Synatschke,^[a]^ Qi Lu,^[a]^ Jiyao Yu,^[a]^ David Y.W. Ng,*^[a]^ Tanja Weil*^[a][b]^

^[a]^ Max Planck Institute for Polymer Research, Ackermannweg 10, D-55128 Mainz, Germany

^[b]^ Max Planck School Matter to Life, Jahnstraße 29, D-69120 Heidelberg, Germany

‡These authors contributed equally to this work

*Corresponding author: david.ng@mpip-mainz.mpg.de, weil@mpip-mainz.mpg.de

**Materials**

Dibenzofulvene (DBF) was purchased from BLD Pharm. Control peptides **P2** and **P3** were purchased from GenScript (≥ 95% purity) and their purity confirmed by HPLC-MS (Fig. S1). Fmoc-Rink amide MBHA resin and all standard fluorenylmethyloxycarbonyl (Fmoc)-protected amino acids were obtained from Novabiochem^®^. HEPES, activator base OxymaPure^®^, trifluoroacetic acid (TFA) and piperidine were purchased from Carl Roth. Activator N,N’ diisopropylcarbodiimide (DIC) was obtained from Sigma-Aldrich. N,N-Dimethylformamide (DMF for peptide synthesis), diethyl ether and dimethylsulfoxide (DMSO) were purchased from Acros Organics. Acetonitrile (MeCN) was obtained from Thermo Fisher Scientiﬁc. Nile Red, 6-methoxy-2-naphthaldehyde (MND) and 4-nitrophenyl acetate (PNPA) were purchased from TCI. Uranyl acetate was purchased from Merck. If not stated otherwise, commercially obtained chemicals were utilised without further purification.

**Methods**

**Synthesis of peptide**

**P1** was synthesised as similarly described previously.^[14]^ Briefly: Fmoc-Rink amide MBHA resin (0.25 mmol scale) was swollen in 5 mL at RT for 1 h. The synthesis was carried out from C- to N-terminus using the Liberty Blue Automated Microwave Peptide Synthesiser by CEM Corporation. After transferring the resin to the reaction chamber, residual DMF was drained and the resin swollen in DMF for 20 sec. (i) For Fmoc-removal, solvent draining was followed by the addition of 20% v/v piperidine in DMF. Heating cycles with microwaving at 155W for 15 s at 75 °C and 30W, 50 s and 90 °C were applied after which the reaction solution was drained. (ii) The resin was then washed three times with DMF before amino acids (5 equ. relative to resin loading), OxymaPure^®^ (10 equ.) and DIC (5 equ.) dissolved in DMF were added and the coupling solution heated to 75°C for 15 s at 155W followed by a 90°C for 50 s at 30W treatment. After draining, steps (i) and (ii) were repeated. To afford the final peptide sequence Fmoc-HLRLKLK-NH_2_ (**P1**), the final deprotection step was omitted. After synthesis, the peptide was cleaved off the resin using a 10 mL cleavage cocktail containing TFA, triisopropylsilane, MQ (95%/2.5%/2.5% v/v) for 2 hours at RT. The resin was washed thrice with 2 mL of pure TFA and the solution reduced under HV. The crude was dissolved in MeCN/MQ (30%/70% v/v) containing 0.1% TFA, filtered and purified by prep RP-HPLC on a Phenomenex Gemini 5 μM NX-C_18_ 110 Å 150 × 30 mm using a linear gradient at 25 mL/min on a setup by Shimadzu. The eluate was collected, pooled and freeze dried under HV. The purity was assessed by HPLC-MS using a Kinetex EVO C_18_ 100 Å LC column (50 × 2.1 mm, 2.6 μm) at 214 nm (Fig. S1). The obtained results were processed using LabSolutions by Shimadzu and Origin Pro^®^2023b.

**Experimental techniques**

**Assembly/Gelation**

Peptide assemblies were prepared by dissolving 2.5 mM of peptide (100 mM stock solution in DMSO) and 2.5 mM of Fmoc-Gly (100 mM stock solution in DMSO) in HEPES buffer (100 mM pH 8) and keeping the DMSO content (20% v/v) constant.^[35]^ Inside the 1.5 mL glass vial, the solution was heated for 30 sec at 70°C under shaking until full dissolution followed by equilibration at RT. In control experiments lacking one of the constituents, the respective portion was replaced by DMSO.

**Transmission Electron Microscopy Imaging (TEM)**

Peptide assemblies were diluted to 500 µM using 20%/80% v/v DMSO/HEPES buffer 100 mM pH 8. 5 µL of the solution was applied on the active surface of a formvar/carbon-film coated copper grid (300 mesh), incubated at RT for 10 min and excess solution was removed using filter paper.^14^ Afterwards, the grids were stained for 2.5 min using 10 μL of a 4% uranyl acetate solution. After removal of excess solution, the grids were washed thrice with MQ and air dried overnight. The data was recorded on a JEOL 1400 TEM microscope at a voltage of 120 kV. The obtained data was processed in Fiji ImageJ.

**Liquid Chromatography-Mass Spectrometry**

For compound analysis, a LC-MS 2020 by Shimadzu equipped with a Kinetex 2.6 µm EVO *C_18_* 100 Å column (size: 50 × 2.1 mm) was used. For the mobile phase ACN and H_2_O were utilised, each acidified with 0.1 % formic acid. All measurements were started with 5 % ACN while its concentration was linearly increased to 95 % in 20 min. The analysed samples were dissolved in ACN, MeOH and/or H_2_O. The collected data was processed using LabSolutions by Shimadzu and PowerPoint by Microsoft Corporation.

**NMR-Spectroscopy**

^1^H-NMR-measurements was conducted in an AVANCE 400-spectrometer by Bruker at 278 K. Chemical shifts δ of 1H spectra are given in parts per million (ppm) from high to low frequency. For the measurements, D_2_O and DMSO-d6 80%/20% v/v with 100 mM HEPES pH 8 was utilised. The data was processed with MestReNova 14.2.0 by Mestrelab Research.

**Rheology**

The rheological characterisation was performed using a DHR3 rheometer (TA Instruments), which was equipped with a temperature controller and a solvent trap to prevent hydrogel dehydration. The experiments were conducted using an 8 mm parallel-plate geometry, with samples of 35 µL volume, resulting in a gap size ranging from 0.55 to 0.65 mm. The mechanical properties of the hydrogels were characterised at 25 °C. For **Frequency Sweep,** the samples were prepared in the parallel plate geometry and incubated for the desired time. Frequency sweeps spanning from 0.1 Hz to 100 Hz were then executed at a fixed strain of 1% at 25 °C. For **time Sweep,** the linear viscoelastic region was identified at 1% strain and 1 Hz frequency. Consequently, oscillatory time-sweep measurements were carried out at a fixed strain of 1% and a fixed frequency of 1 Hz at 25 °C for a duration of 6 hours with a measurement interval of 5 minutes. The obtained data was processed by Origin Pro^®^2023b.

**Confocal Laser Scanning Microscopy (CLSM)**

Series of samples at different time intervals were incubated with Nile Red (30 µM) for 10 min in the dark at RT prior to measurement. 10 µL of solution were applied to a 15-well IBIDI® chamber and fluorescence was detected by a STELLARIS 8 Leica DMi8 microscope (Leica Microsystems) using a HyD S detector (λ_ex_ = 530 nm, λ_em_ = 550 nm – 750 nm), a 93x glycerol immersion objective and an Airy unit of 1. Images were processed by LAS X from Leica and scale bars were added in Fiji ImageJ. All samples were fluorescence background subtracted. For better visualisation, the samples containing fibres were maximally projected (z-stack size: 0.33 µm).

**Atomic Force Microscopy (AFM)**

Atomic force microscopy (AFM) (Park NX20 and Bruker Dimension ICON) was employed to characterise the morphology of the system. Briefly, samples at different time intervals were diluted to 250 µM and air-dried prior casting on freshly cleaved silicon wafer. Imaging was performed under tapping mode using a cantilever with a resonance frequency of 285 kHz and a spring constant of 42 N·m⁻¹. The resulting AFM images were further processed and analysed with Gwyddion 2.63.

**Fluorescence study**

All experiments were conducted using a Greiner 384 flat black well plate on a SPARK 20M microplate reader machine by Tecan Group Ltd. Briefly, series of samples at different time intervals were incubated with 30 µM of Nile Red for 10 mins prior measurements. Fluorescence scans were carried out at λ_ex_: 559 nm. The collected data was processed by Origin Pro^®^2023b.

**Turbidity kinetics**

Turbidity measurements were conducted in 384 flat transparent UV-star well plates from Greiner. 50 µL of solution was added to each well, the chamber sealed and the turbidity measured at 600 nm by a SPARK 20M microplate reader machine by Tecan Group Ltd. The collected data was processed by Origin Pro^®^2023b.

**HPLC kinetics**

HPLC kinetics were conducted by preparing 50 µL peptide assembly samples for each time stamp as outlined previously. The reaction solution was quenched and diluted simultaneously with 1200 µL MeCN/MQ (50%/50% v/v) containing 0.1% TFA yielding a 200 µM quenched sample. The evolution of chemical species was traced by injecting 50 µL on an Atlantis T3 column (4.6 × 100 mm, 5 μm) with a flowrate of 1 mL/min following a linear gradient of MeCN. For the calculation of the relative peak areas in percent, the following equation was utilised:

1. ${Ratio}_{x}\left( \% \right)= \left( \frac{A_{x}}{A_{x}{+ A}_{y}} \right)\times100$

*(1)* With *A_x_* being the absolute peak area of **Fmoc-Gly** and A_y_ being the absolute peak area of DBF or vice versa. Signals were detected at 214 nm and processed using Origin Pro^®^2023b.

**Activity measurements**

The activity of the system toward hydrolase activity were monitored in SPARK 20M microplate reader machine (Tecan Group Ltd).^[10]^ Briefly, 0.1 mM of PNPA/PNBA (DMSO stock) for hydrolysis or 0.2 mM NBD-H (fresh, water stock) and 0.4 mM 6 MND (DMSO stock) were added to 0.5 mM **P1/Fmoc-Gly** (diluted with HEPES buffer from 2.5 mM **P1/Fmoc-Gly** system) at different time intervals (5 min, 2 h and 10 h) and transfer it to 384 flat transparent UV-star well plates (path length 0.4 cm). The change of absorption at 400 nm for hydrolysis and 524 nm for C-N condensation were monitored overtime to track the generation of the product, para nitro phenol (ε at 400 nm is 8100 M^-1^cm^-1^, Figure S15) and hydrazone adduct (ε at 524 nm is 4625 M^-1^cm^-1^, Figure S17) respectively. The collected data was processed by Origin Pro^®^2023b.


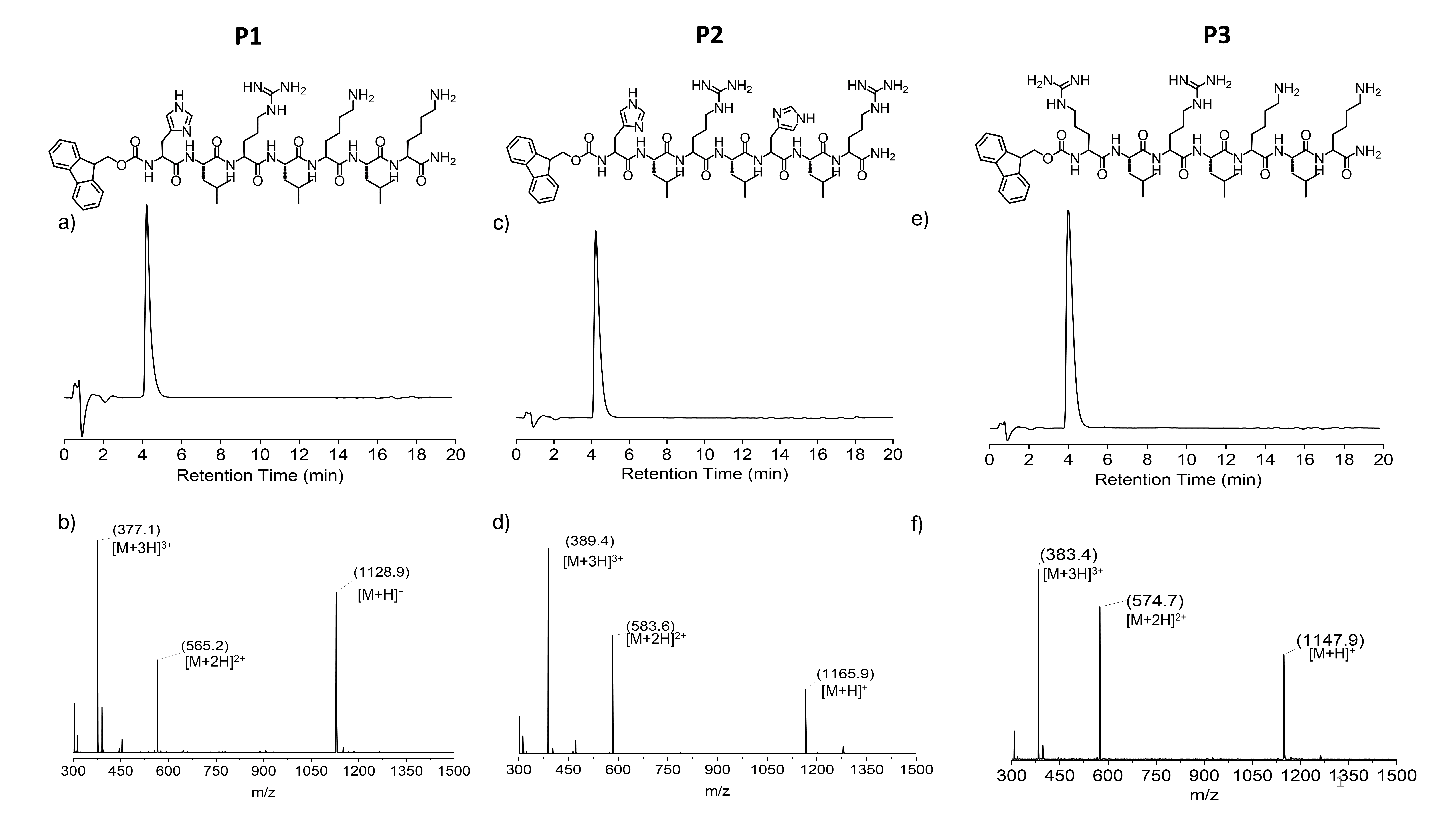


Figure S 1. LCMS analysis of peptides. (a) LC trace of **P1**. (b) Masses corresponding to the main peak. calc.: m/z = 1129.4 [M + H]^+^, meas.: m/z: 1128.9 [M + H]^+^. (c) LC trace of **P2**. (d) Masses corresponding to the main peak. calc.: m/z = 1166.4 [M + H]^+^, meas.: m/z: 1165.9 [M + H]^+^. (e) LC trace of **P3**. (f) Masses corresponding to the main peak. calc.: m/z = 1148.5 [M + H]^+^, meas.: m/z: 1147.9 [M + H]^+^. LC traces were recorded at 214 nm.


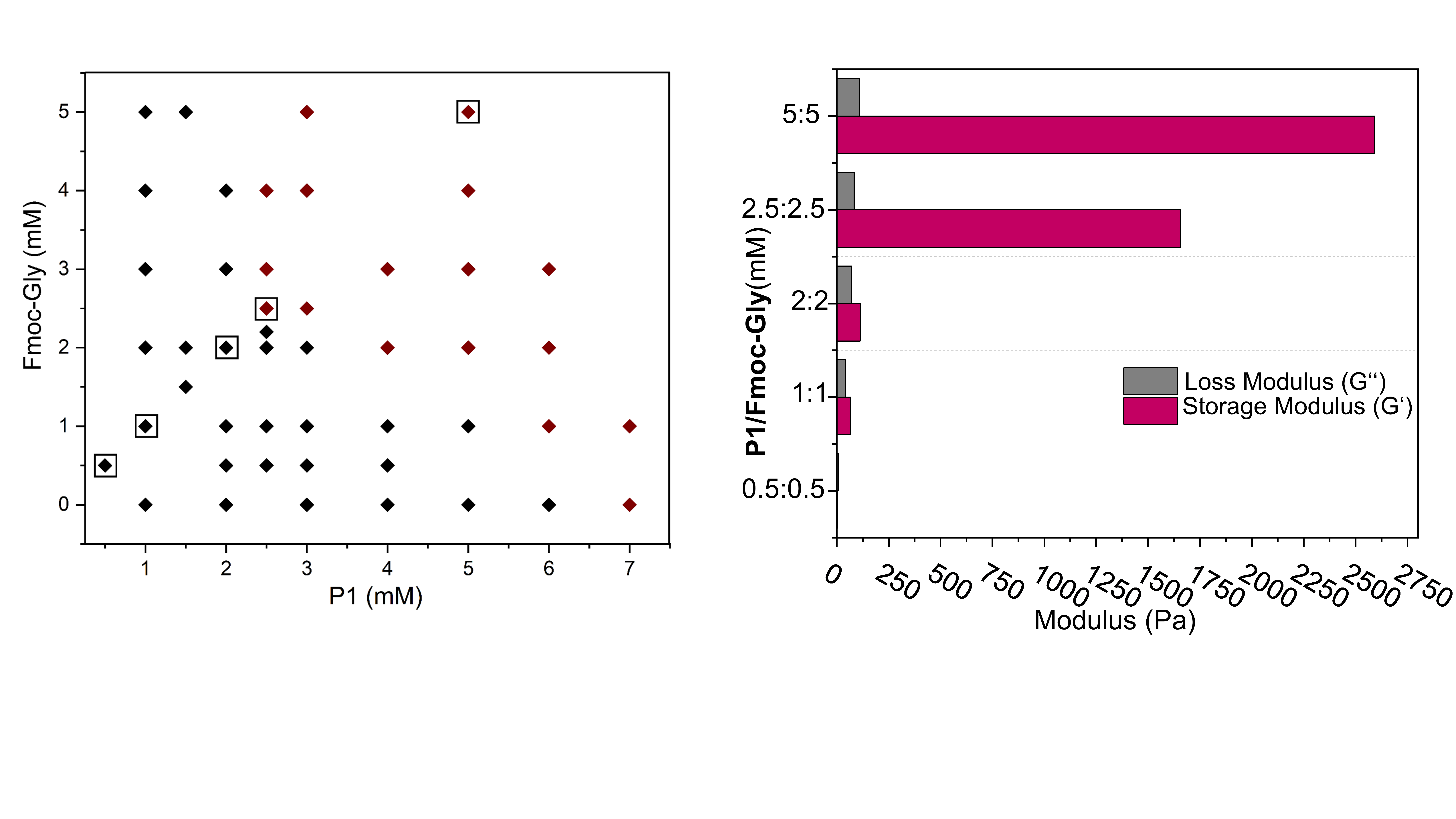


Figure S 2. Self-supporting gel (red dots) and viscous sol (black dots) by **P1/Fmoc-Gly** system (left panel). Rheology of square labelled points shows the viscoelastic gel characteristics in the bar diagram (right panel).


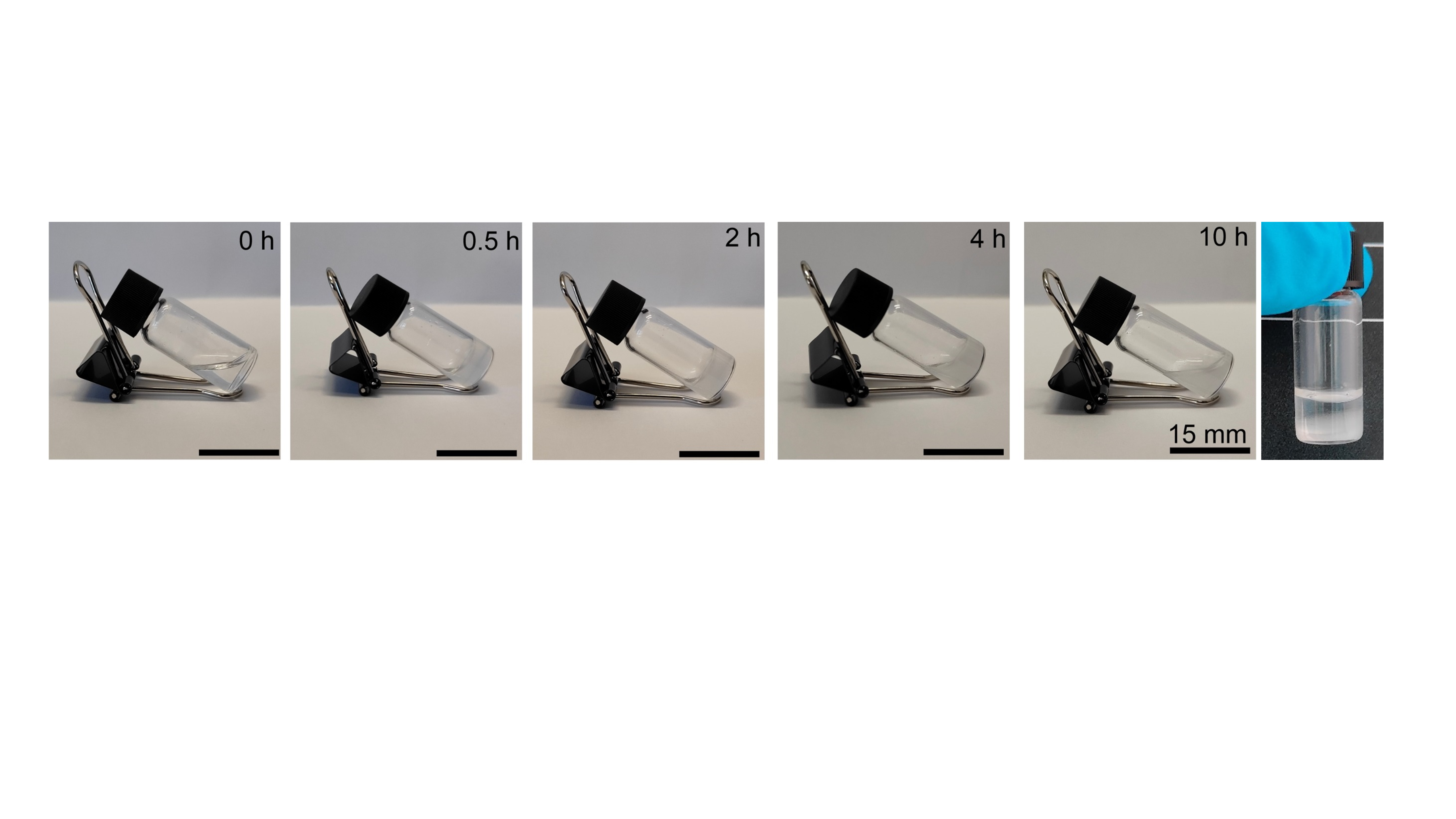


Figure S 3. Macroscopic bulk properties of the **P1/Fmoc-Gly** hydrogel (2.5 mM each) showing the sol-gel-sol transition. Extreme right picture with black background shows phase separation.


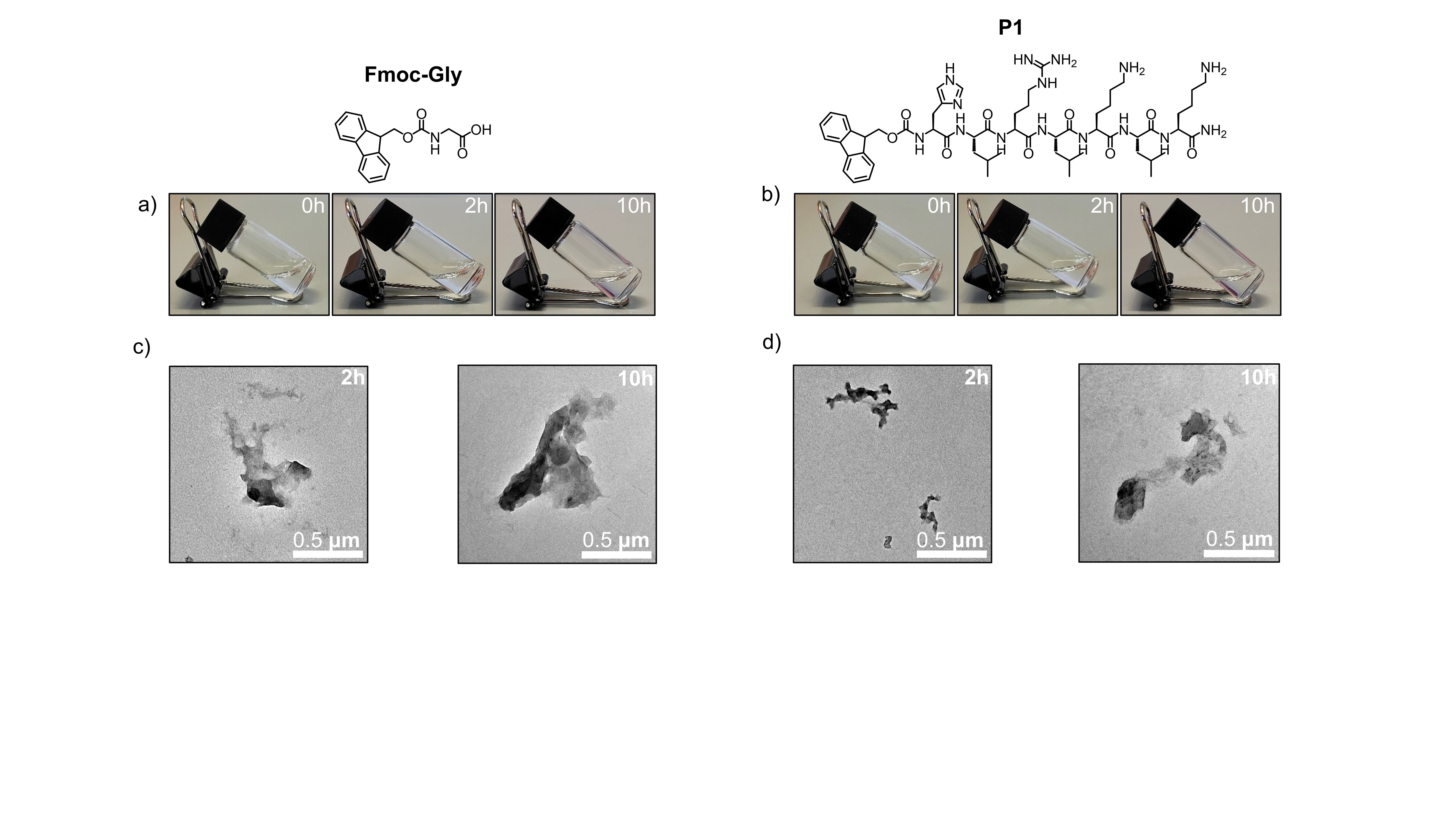


Figure S 4. Characterisation of individual **Fmoc-Gly** and **P1** control samples. (a) Solution state of **Fmoc-Gly** (2.5 mM) over time. (b) Solution state of **P1** (2.5 mM) over time. (c) TEM micrographs of **Fmoc-Gly** (2.5 mM stock) after 2 h and 10 h. (d) TEM micrographs of **P1** (2.5 mM stock) after 2 h and 10 h. Final TEM concentration after dilution: 0.5 mM.


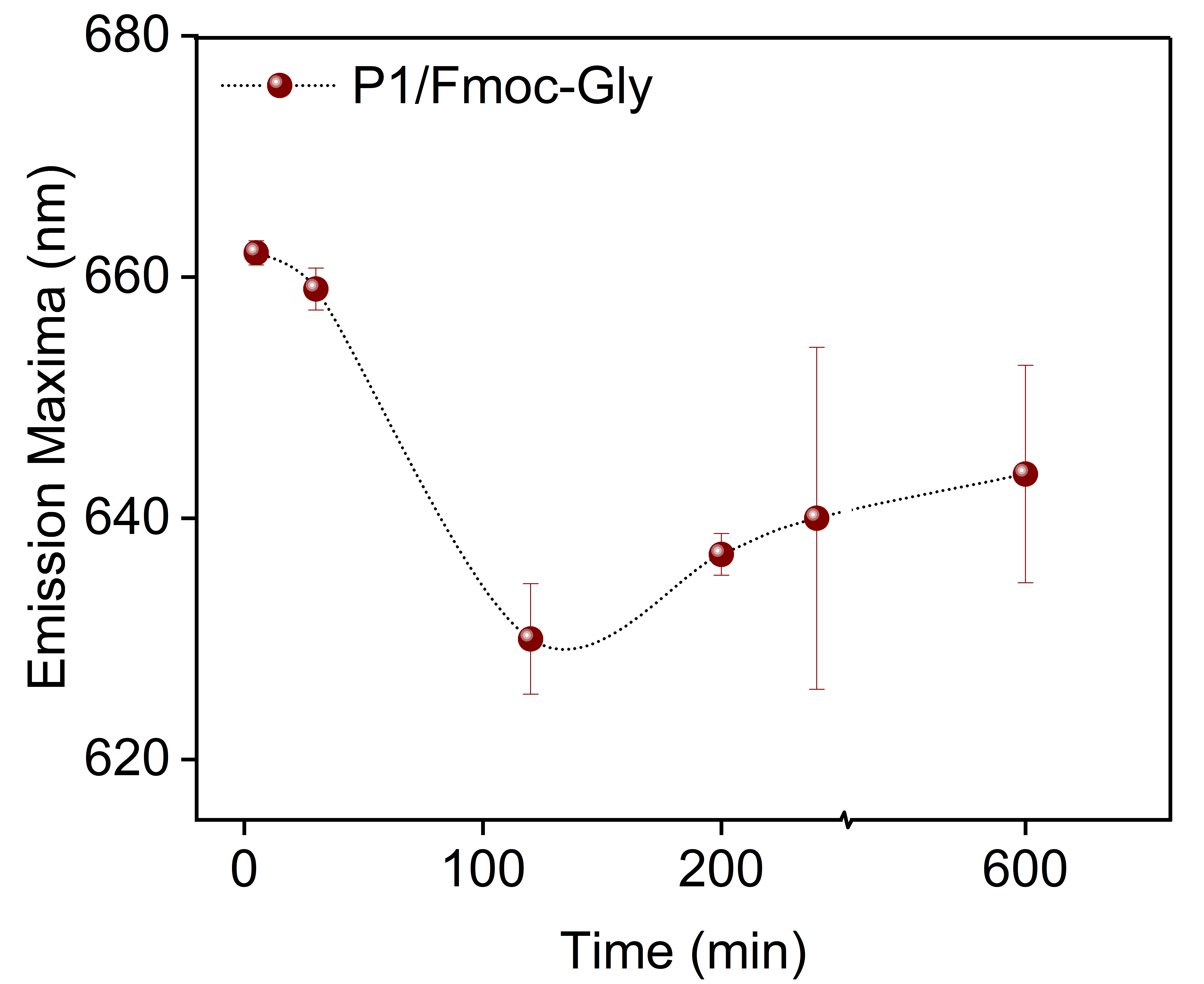


Figure S 5. Transient changes of emission maxima of NR in presence of **P1/Fmoc-Gly** (2.5 mM each). Error bars correspond to standard deviations of triplicates. [NR]=30 μM.


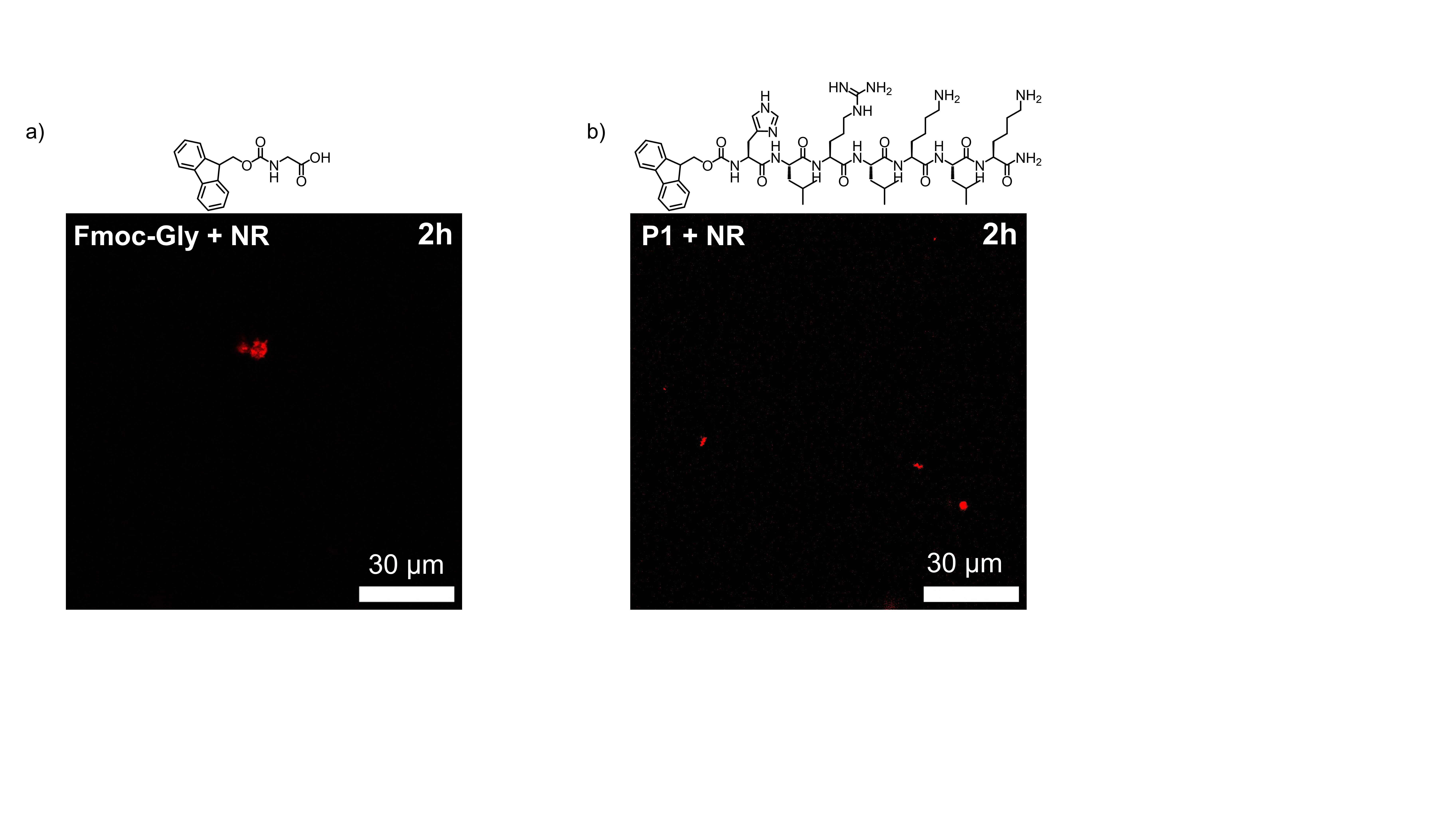


Figure S 6. Confocal laser scanning microscopy micrographs of (a) **Fmoc-Gly** (2.5 mM); (b) **P1** (2.5 mM) after 2 h of incubation. [NR]=30 μM.


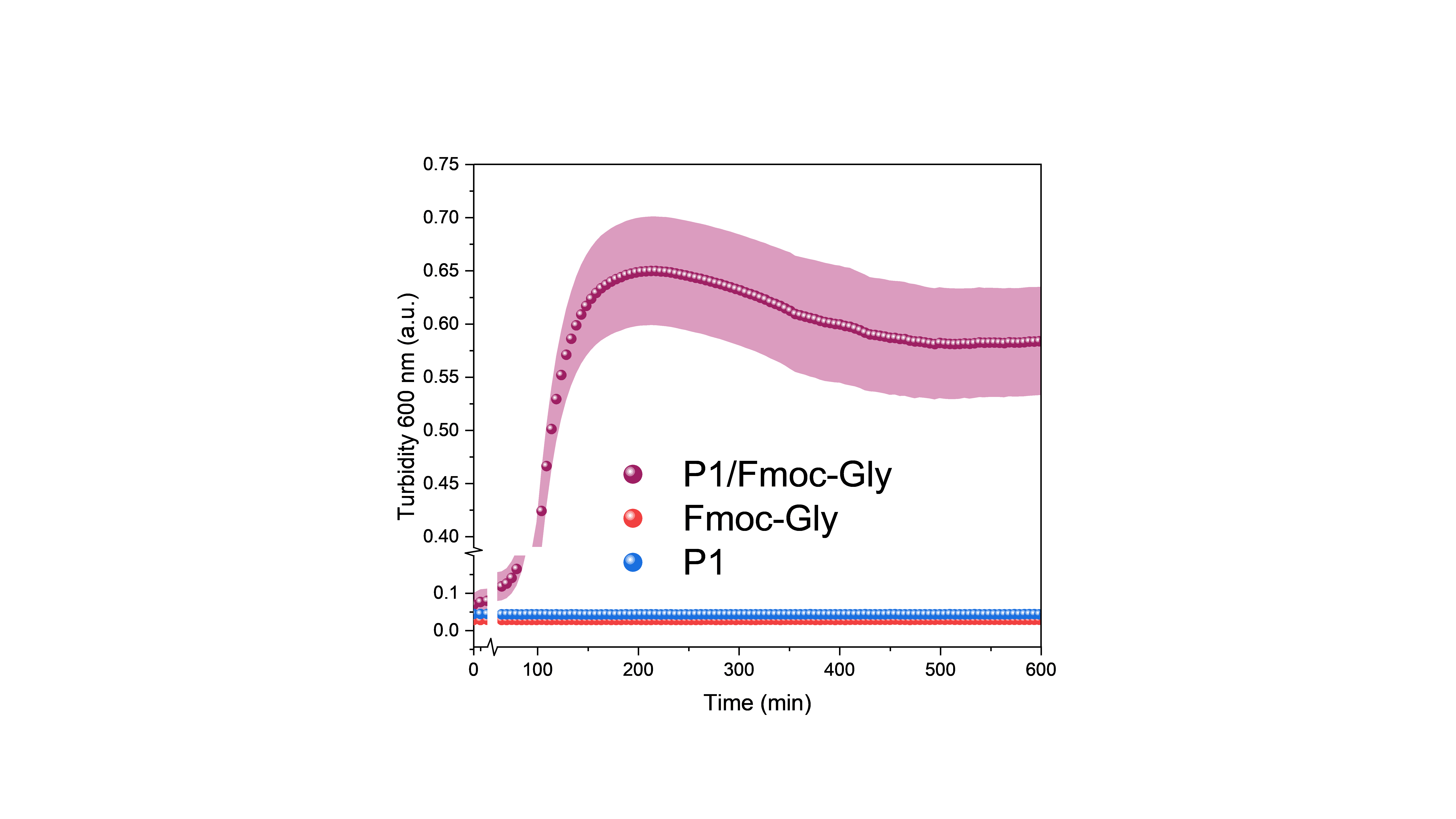


Figure S 7. Turbidity measurements at 600 nm with **P1/Fmoc-Gly** (2.5 mM each) system and control samples **Fmoc-Gly** and **P1** alone (2.5 mM each). Error bars correspond to standard deviations of triplicates.


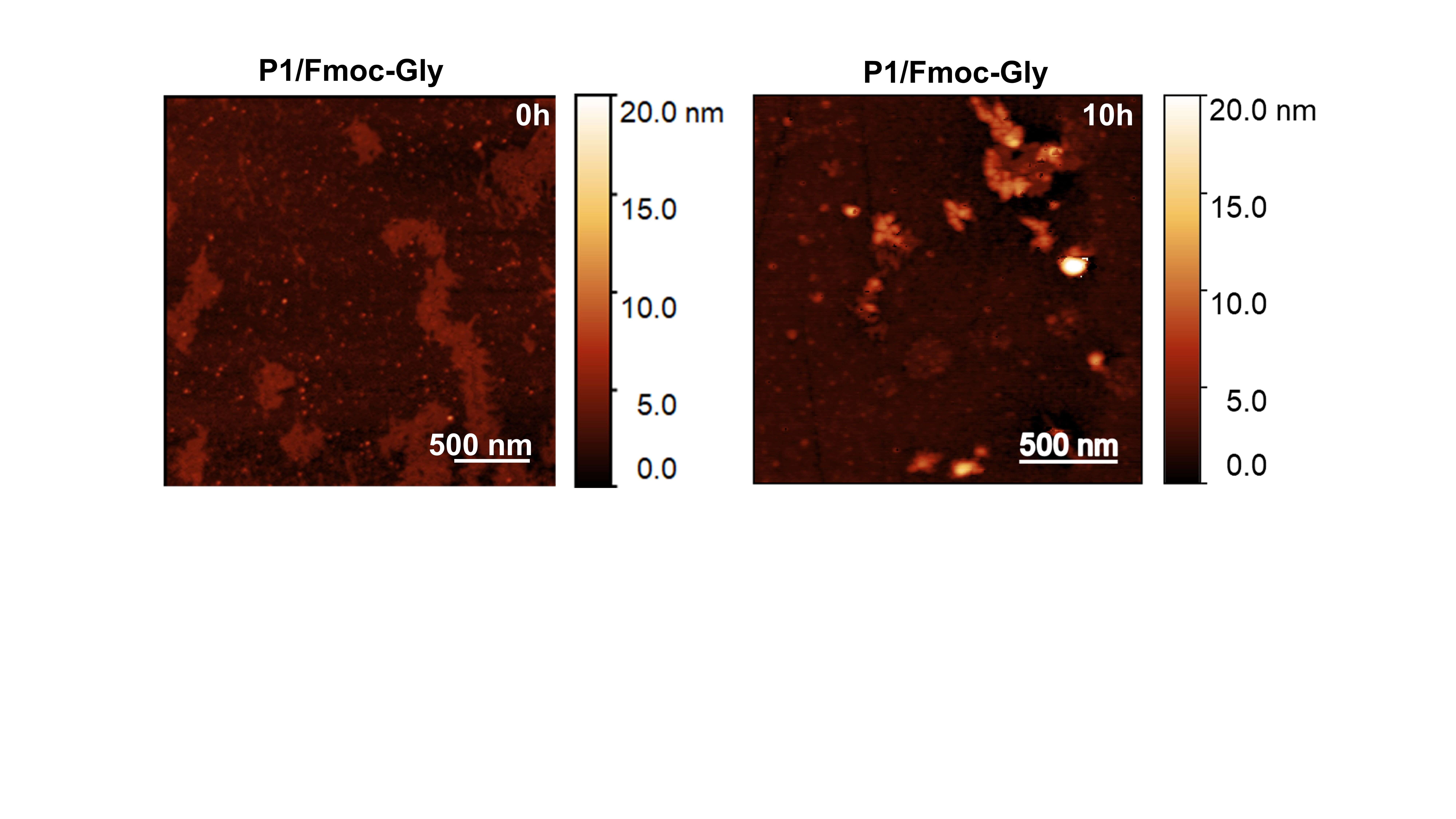


Figure S 8. AFM micrographs of **P1/Fmoc-Gly** system (2.5 mM) at initial t=0 h and final stage of assembly t=10 h. Final AFM concentration after dilution: 0.25 mM.


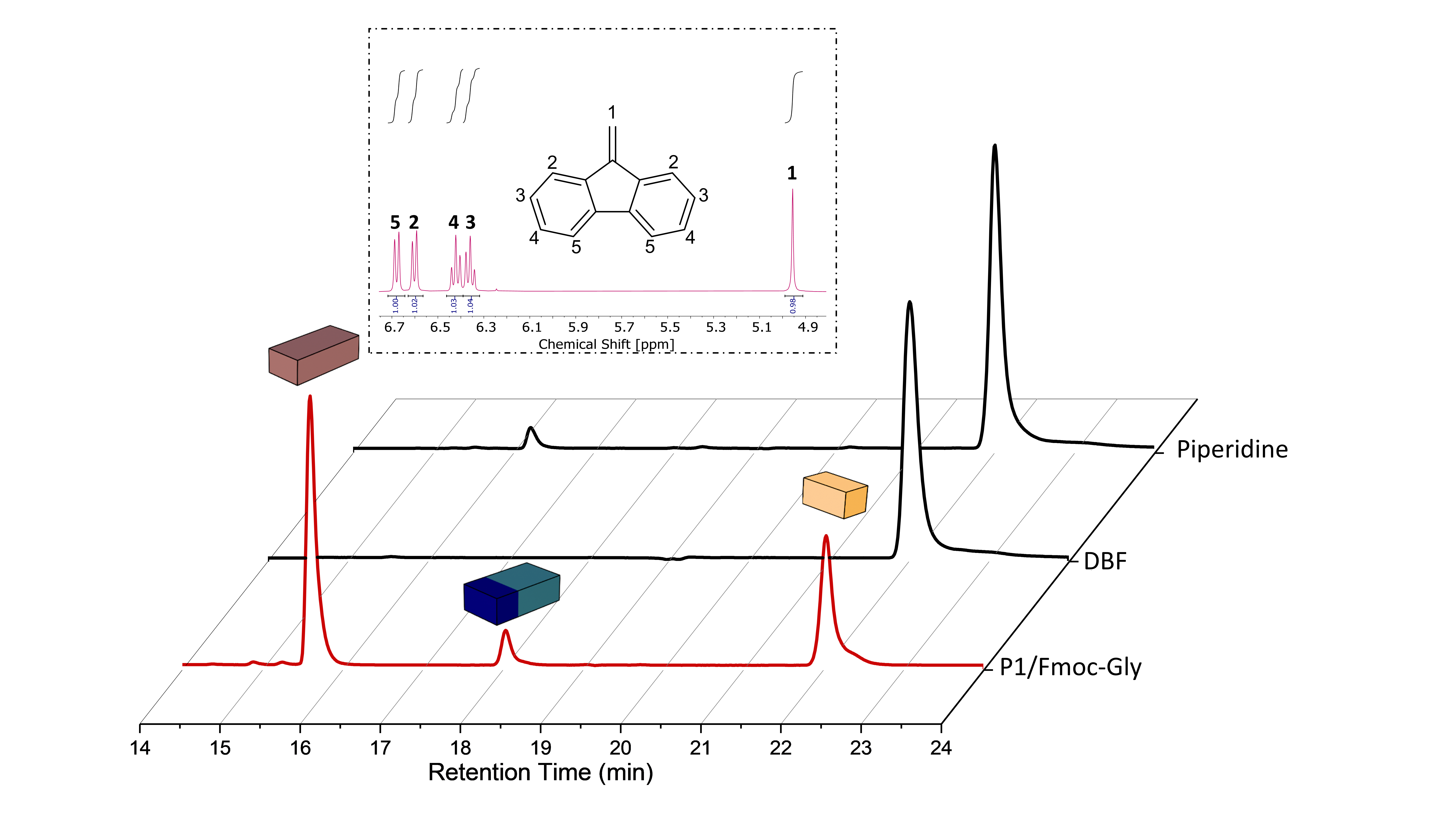


Figure S 9. DBF product formation confirmation. HPLC traces of **P1/Fmoc-Gly** sample after 10 h (red line), commercially purchased DBF and piperidine-treated (5 mM) **Fmoc-Gly** sample showing the same product retention time. t_R_ = 22.0 min. Insert shows ^1^H-NMR of commercially purchased DBF in the same buffer conditions (using deuterated solvents) as the **P1/Fmoc-Gly** system.


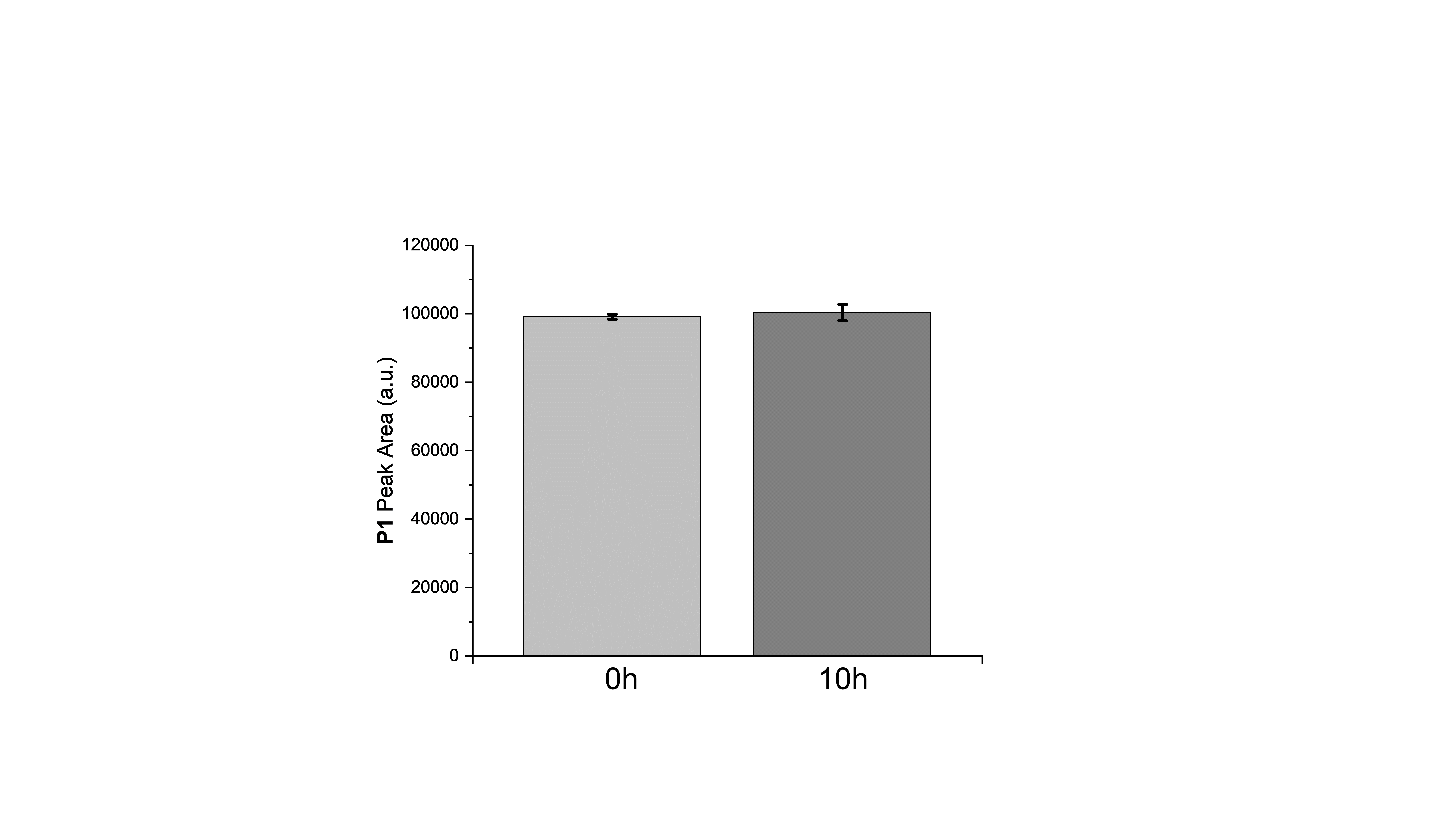


Figure S 10. Bar diagram of **P1** peak area over time in the **P1**/**Fmoc-Gly** system showing no significant reduction of signal within 10 h (**P1** concentration 2.5 mM). Error bars show standard deviations from triplicates.


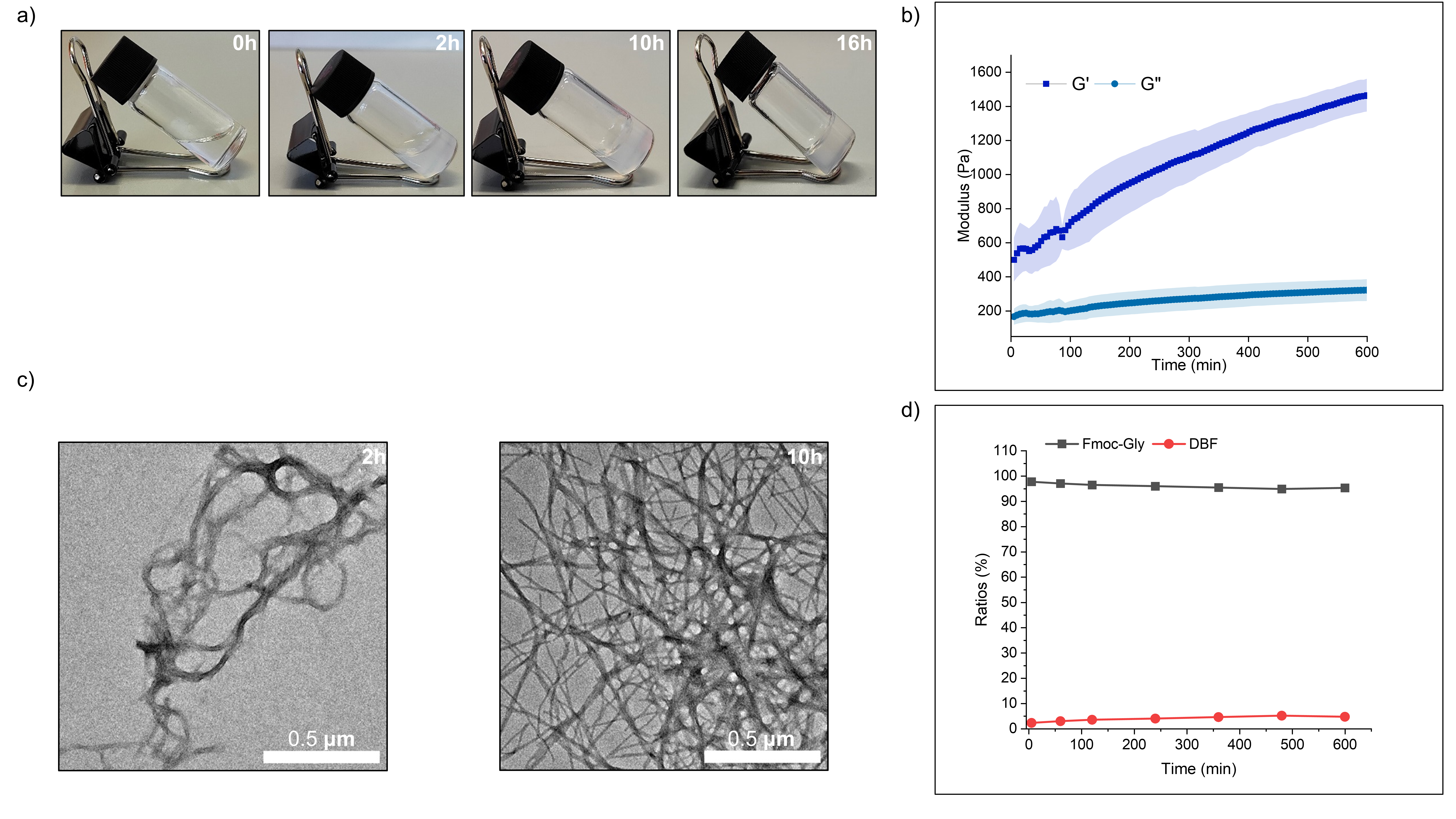


Figure S 11. Characterisation of the **P2/Fmoc-Gly** system. a) Sol-to-gel transition of **P2/Fmoc-Gly** system over time. b) Rheology time sweep experiments showing the gradual stiffening of the gel. Experiments were done at a constant frequency of 1 Hz and a strain of 1 %. Error bars show standard deviations from triplicates. c) Time-dependent TEM micrographs of **P2/Fmoc-Gly** assembled structures. d) Relative ratios of DBF and **Fmoc-Gly** over time as determined by HPLC showing negligible DBF formation. Standard deviations shown as error bars correspond to triplicates (too small to visualise). All experiments were done at a concentration of 2.5 mM for **P2** and **Fmoc-Gly**.


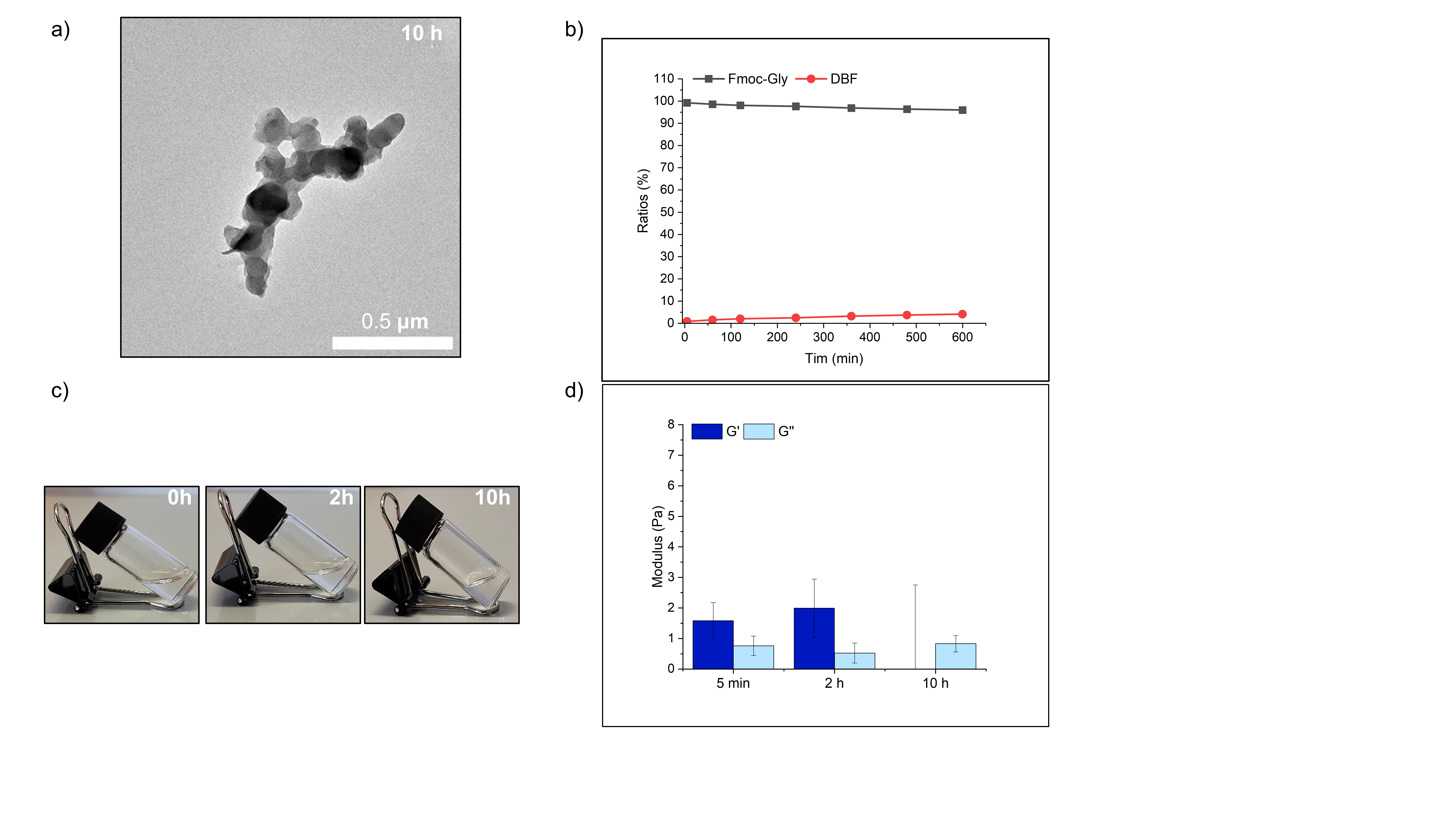


Figure S 12. Characterisation of the **P3/Fmoc-Gly** system. a) TEM micrographs of **P3/Fmoc-Gly** after 10 h. b) Time-dependent evolution of **Fmoc-Gly** and DBF in **P3/Fmoc-Gly** showing no significant increase in DBF formation over 10 h as determined by HPLC. Standard deviations shown as error bars correspond to triplicates (too small to visualise). c) Solution state of **P3/Fmoc-Gly** over time. d) Rheology experiment probing the mechanical properties of the sample at a frequency of 1 Hz and a strain of 1 %. For 10 h time stamp, no reliable G’ could be determined as the solution was too liquid. All experiments were done at a concentration of 2.5 mM for **P3** and Fmoc-Gly.


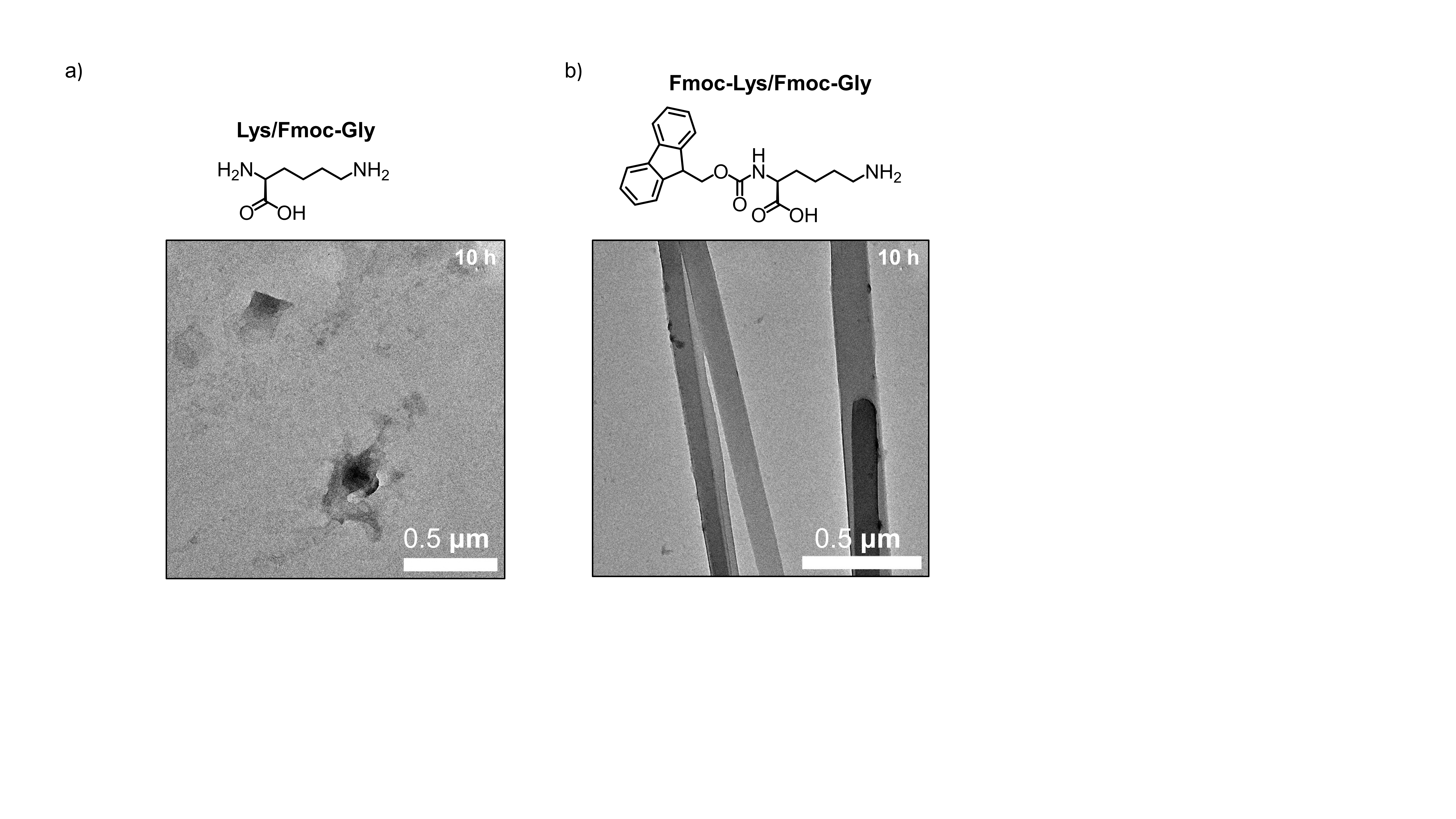


Figure S 13. TEM micrographs of the a) **Lys/Fmoc-Gly** and b) **Fmoc-Lys/Fmoc-Gly** system after 10 h of incubation. All experiments were done at a concentration of 2.5 mM for each component.


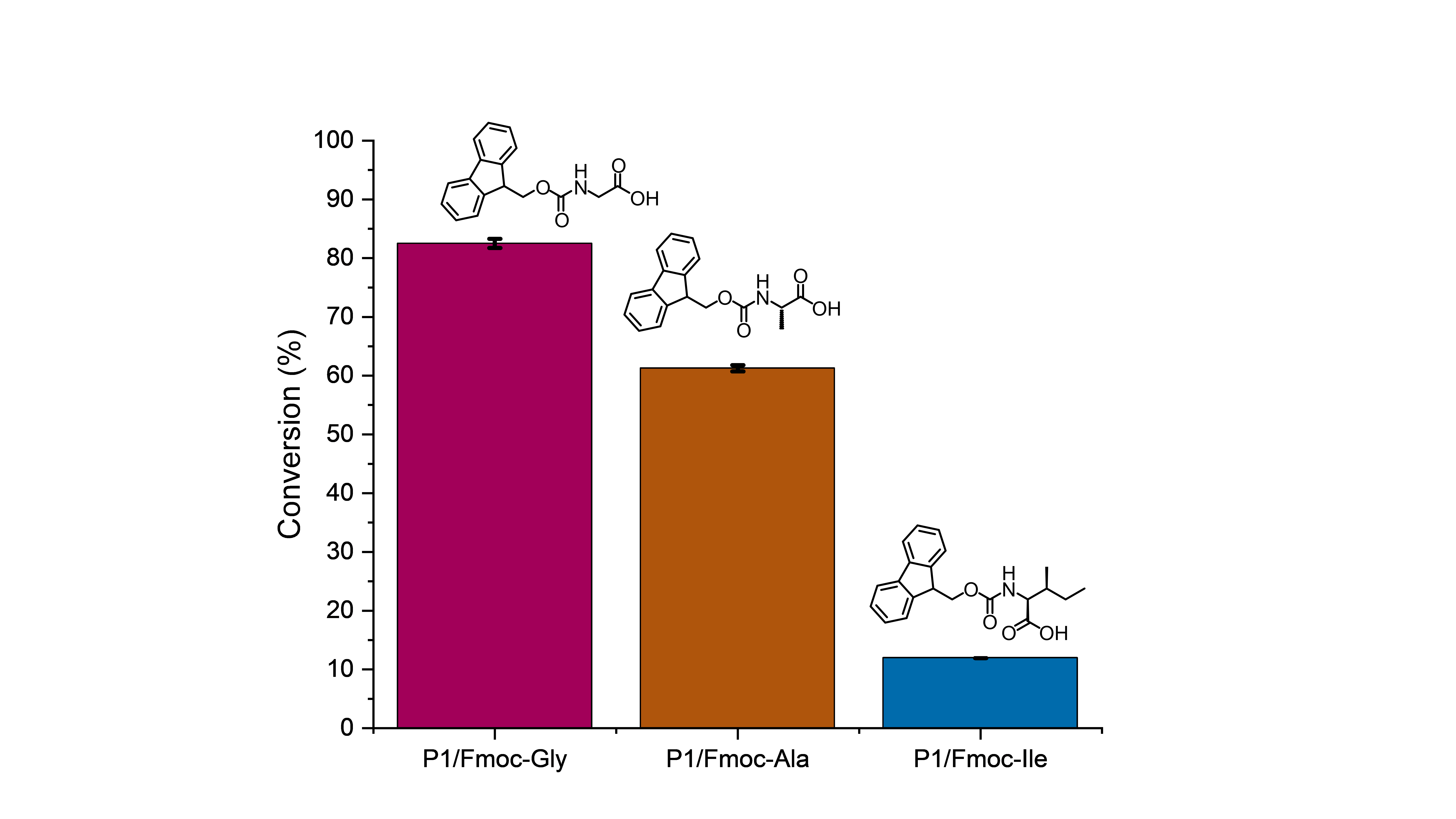


Figure S 14. Conversion of different Fmoc-protected substrates into DBF after 10 h of incubation with **P1** as determined by HPLC. Error bars show standard deviations from triplicates. All experiments were done at a concentration of 2.5 mM for each component.


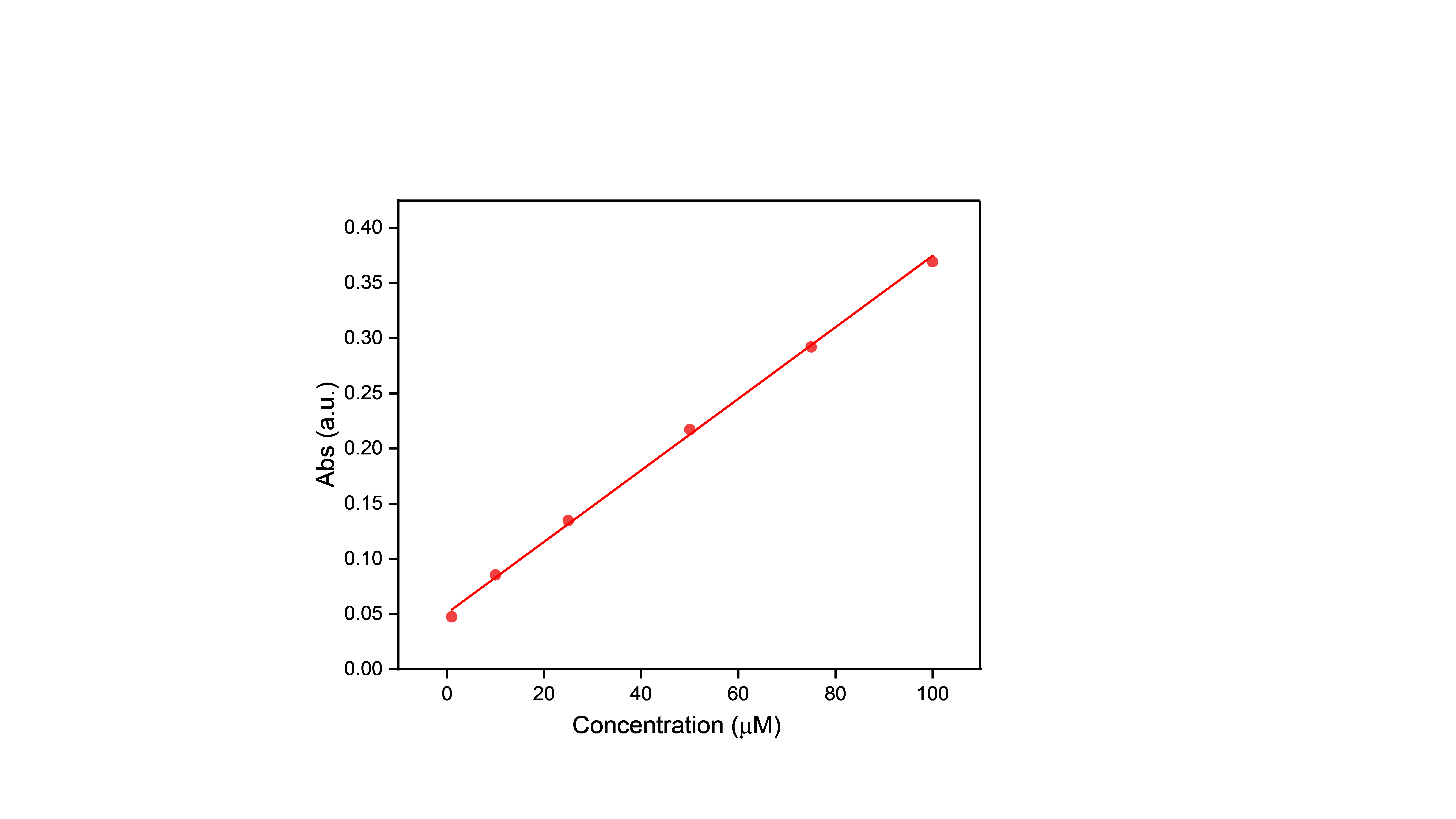


Figure S 15. Standard plot of PNP from UV-Vis (at 400 nm).


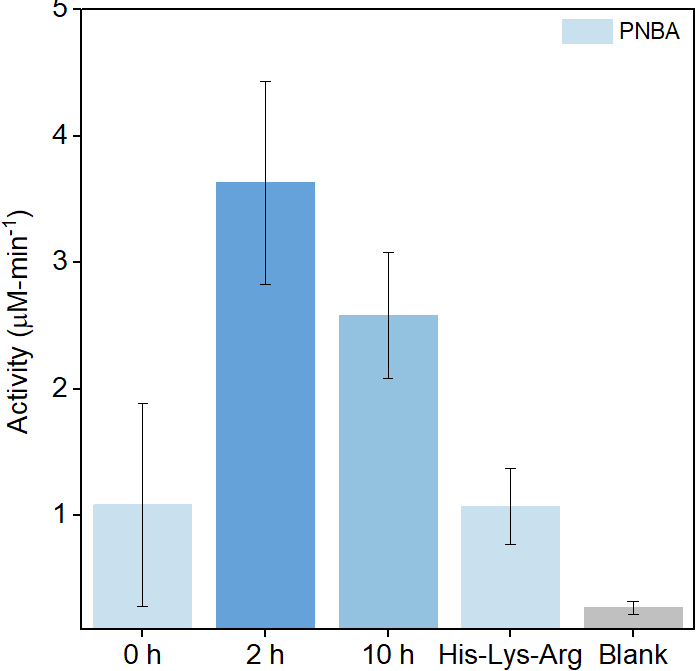


Figure S16: Bar diagram showing the comparison of initial rates towards PNBA hydrolysis. [**P1/Fmoc-Gly** ]=0.5 mM (assembly concentration: 2.5 mM). [His]=[Arg]=0.5 mM; [Lys]=1 mM.


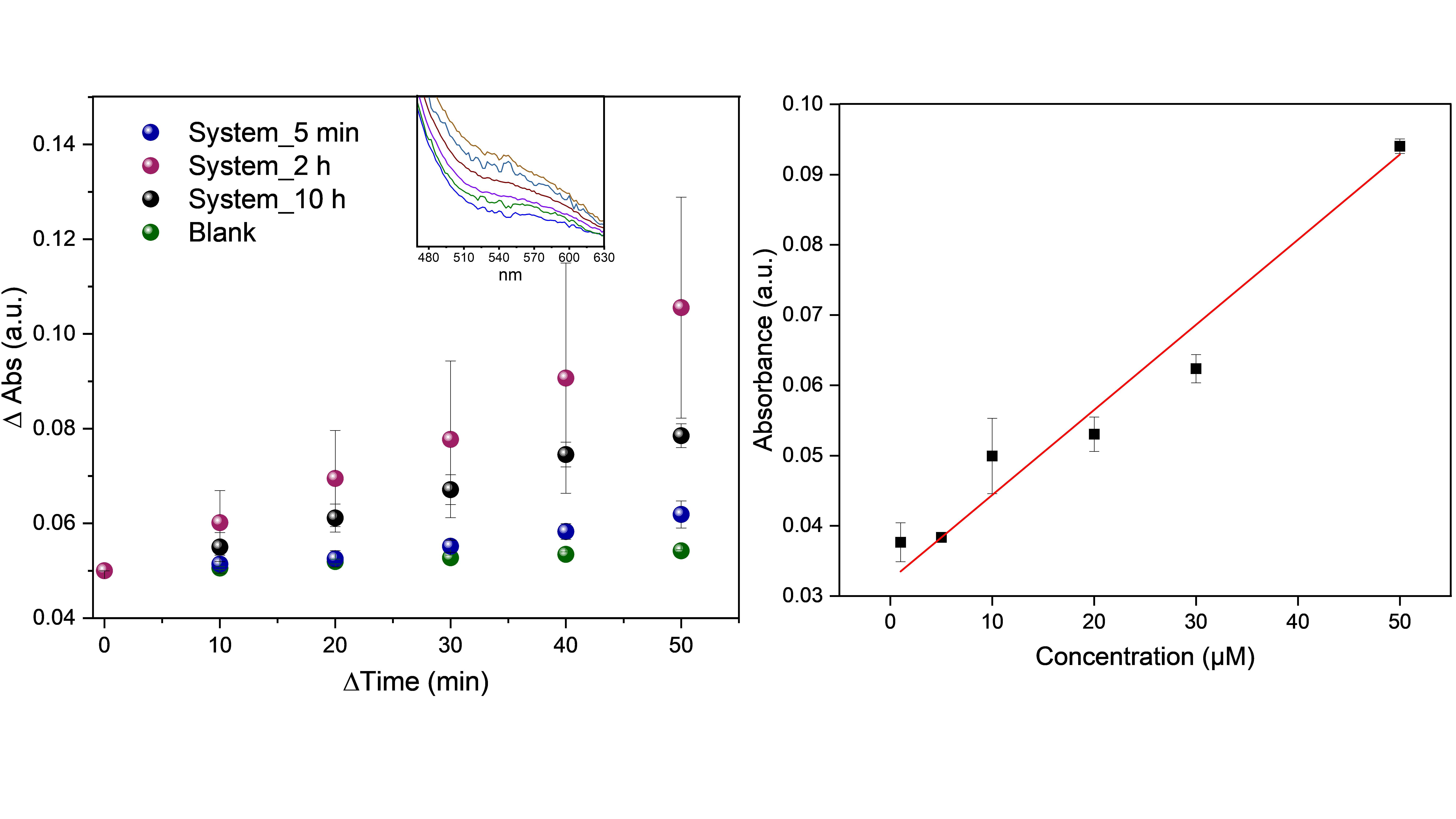


Figure S 17. Left panel shows time course kinetic profile of NBD-H and 6 MND condensation monitored at 524 nm, Inset shows increase of broad absorption band, indicating the adduct formation. Right panel shows standard plot of adduct between NBD-H and 6 MND from UV-Vis (at 524 nm). Error bars correspond to standard deviations from triplicates.


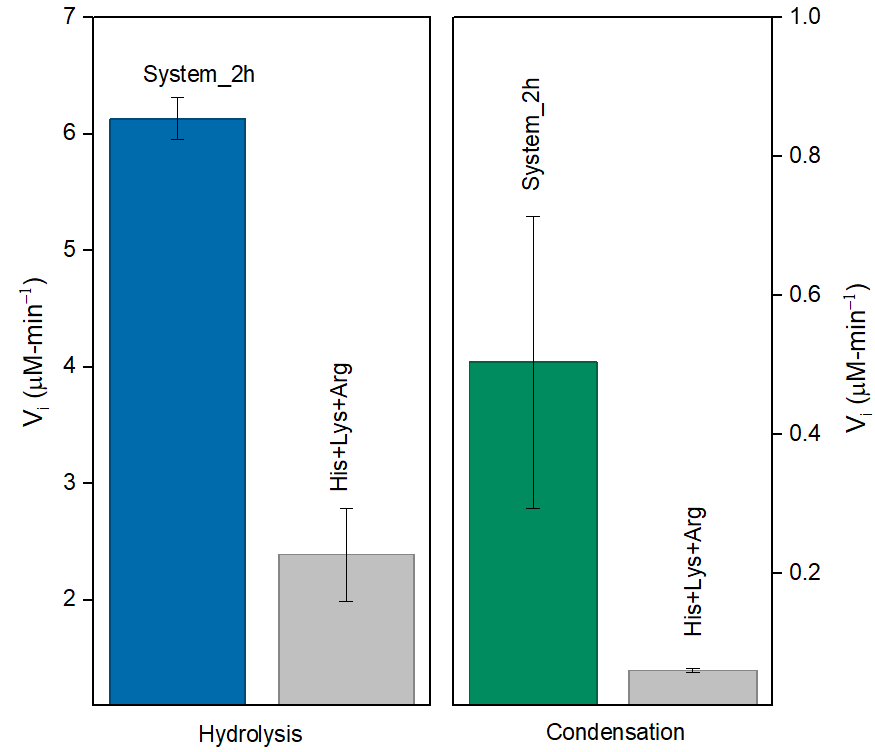


Figure S 18: Comparison of catalytic abilities of **P1/Fmoc-Gly** at 2 h for PNPA hydrolysis and condensation compared to free flowing solution of His, Lys and Arg. [**P1/Fmoc-Gly**]=0.5 mM (assembly concentration: 2.5 mM). [His]=[Arg]=0.5 mM; [Lys]=1 mM.
